# Bridging the diversity gap: Analytical and study design considerations for improving the accuracy of trans-ancestry genetic prediction

**DOI:** 10.1016/j.xhgg.2023.100214

**Published:** 2023-06-15

**Authors:** Ozvan Bocher, Arthur Gilly, Young-Chan Park, Eleftheria Zeggini, Andrew P. Morris

**Affiliations:** 1 ITG, Helmholtz Zentrum München, Munich, Germany; 2 Technical University of Munich, Munich, Germany; 3 Klinikum Rechts der Isar, Munich, Germany; 4 Centre for Genetics and Genomics Versus Arthritis, Centre for Musculoskeletal Research, University of Manchester, Manchester, UK

**Keywords:** polygenic scores, GWAS, trans-ancestry, complex traits

## Abstract

Genetic prediction of common complex disease risk is an essential component of precision medicine. Currently, genome-wide association studies (GWASs) are mostly composed of European-ancestry samples and resulting polygenic scores (PGSs) have been shown to poorly transfer to other ancestries partly due to heterogeneity of allelic effects between populations. Fixed-effects (FETA) and random-effects (RETA) trans-ancestry meta-analyses do not model such ancestry-related heterogeneity, while ancestry-specific (AS) scores may suffer from low power due to low sample sizes. In contrast, trans-ancestry meta-regression (TAMR) builds ancestry-aware PGS that account for more complex trans-ancestry architectures. Here, we examine the predictive performance of these four PGSs under multiple genetic architectures and ancestry configurations. We show that the predictive performance of FETA and RETA is strongly affected by cross-ancestry genetic heterogeneity, while AS PGS performance decreases in under-represented target populations. TAMR PGS is also impacted by heterogeneity but maintains good prediction performance in most situations, especially in ancestry-diverse scenarios. In simulations of human complex traits, TAMR scores currently explain 25% more phenotypic variance than AS in triglyceride levels and 33% more phenotypic variance than FETA in type 2 diabetes in most non-European populations. Importantly, a high proportion of non-European-ancestry individuals is needed to reach prediction levels that are comparable in those populations to the one observed in European-ancestry studies. Our results highlight the need to rebalance the ancestral composition of GWAS to enable accurate prediction in non-European-ancestry groups, and demonstrate the relevance of meta-regression approaches for compensating some of the current population biases in GWAS.

## Teaser

We investigate the performance of polygenic scores (PGSs) in predicting complex traits in non-European populations. We show through simulations that the genetic heterogeneity of a trait is a key factor influencing PGS predictions, and we highlight the need to rebalance the ancestral composition of future genetic studies.

## Main text

Genome-wide association studies (GWASs) have greatly improved our understanding of the genetics of human complex traits, with over 415,000 genetic associations described across approximately 5,900 studies to date.^1^ This achievement has been made possible partly through the development of large genotyping and sequencing projects including the UK Biobank,^2^ the TOPMed program,^3^ and the FinnGen study.^4^ Although GWASs have reached unprecedented sample sizes, European-ancestry (EUR) individuals still dominate their ancestry composition. They commonly represent around 80% of sample sizes, a ratio that has not decreased in the last few years.^5^ Human populations differ in linkage disequilibrium patterns, allelic frequencies, and exposures to environmental factors.^6^^,^^7^ As a result, common variants associated with a wide range of complex phenotypes tend to have different effect sizes between populations.^8^^,^^9^^,^^10^ Polygenic scores (PGSs) that aim to predict complex human traits by aggregating observed effects across the genome are built from GWASs and are therefore mainly constructed on EUR samples. Due to the heterogeneous genetic architecture between populations, numerous studies have shown that EUR-based PGSs poorly transfer to non-European-ancestry (non-EUR) populations, especially those at greater genetic distance such as those of African ancestry (AFR).^11^^,^^12^^,^^13^^,^^14^^,^^15^ A recent study even showed that a current schizophrenia PGS is more correlated with the ancestry of the individuals than with the trait itself.^16^ GWASs in non-EUR populations tend to be small if they exist at all, which prevents the development of ancestry-specific (AS) PGSs in those populations. To improve predictions in non-EUR populations, studies from diverse ancestries can be combined through the use of meta-analysis. The most used approach is the fixed-effect model that weights the effect size of a study based on the inverse of the variance, with the assumption that all studies measure the same underlying effect for each variant.^17^ These methods perform poorly when genetic heterogeneity is present among contributing studies, a situation that is more likely when combining data from diverse ancestries than when aggregating GWASs from the same population background.^8^^,^^9^^,^^10^ Random-effect models have been developed to address this issue, but they do not assume any pattern in the heterogeneity between studies and tend to have a limited advantage over fixed-effect meta-analyses.^18^ Several further methods aim to specifically answer the question of PGS transferability to non-EUR populations, including the meta-regression model.^19^^,^^20^^,^^21^ In this framework, axes of genetic variation obtained from principal-component analysis (PCA) on the populations are incorporated in the regression model to represent ancestry-related heterogeneity of the genetic effects, leading to a higher predictive power in under-represented populations.^19^^,^^22^ To date, studies on PGS transferability have mostly focused on evaluating population-specific scores (mainly EUR-based) without extensive evaluation of trans-ancestry PGSs.^11^^,^^12^^,^^13^^,^^14^^,^^23^ Even when trans-ancestry PGSs were evaluated, no extensive assessment considering the genetic architecture of the trait or the impact of the ancestry composition of the sample was performed.^24^ This question is important, as trans-ancestry PGSs have been shown to outperform population-specific PGSs for diverse ancestries, including Japanese^25^ and Latino.^20^ To fill in this gap, we developed a TRans-Ancestry PGS Simulation (traps) procedure to perform a comprehensive assessment of the parameters influencing the transferability of four PGS models in non-EUR populations: an AS PGS (AS), a PGS from a fixed-effect trans-ancestry meta-analysis (FETA), a PGS from a random-effect trans-ancestry meta-analysis (RETA), and PGS from a trans-ancestry meta-regression incorporating ancestry-related heterogeneity (TAMR), implemented in the MR-MEGA software.^19^ In this work, we investigate the relative performance of the four approaches via simulation and application to two complex human traits. We chose to focus here on PGS methods constructed from SNPs that meet a predefined significance threshold and therefore did not consider the most recent methods integrating genome-wide SNPs such as PRS-CSx.^24^ Indeed, the main purpose of this study was not to perform a comprehensive quantitative comparison of available PGS methods but rather to assess parameters influencing PGS transferability and support the need for trans-ancestry studies. We focused on this class of PGSs as it has been shown to be of similar performance as genome-wide PGSs in type 2 diabetes (T2D), one of our disease models,^26^ and because we simulated variants without considering linkage disequilibrium (LD), which is more compatible with PGS built up on significant variants.

**Figure 1:**
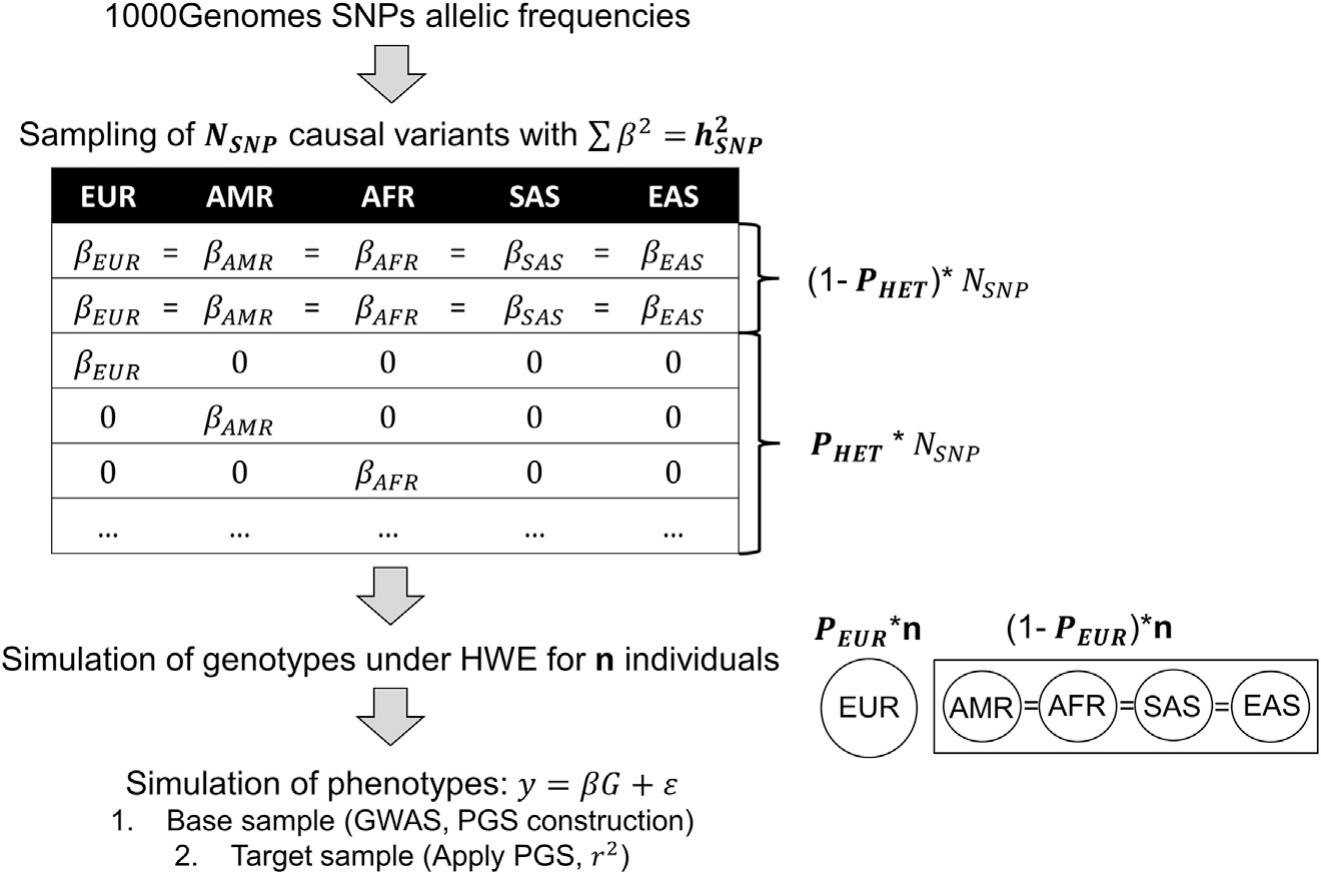
Overview of the traps simulation procedure $N_{SNP}$ corresponds to the number of causal SNPs simulated, $h_{SNP}^{2}$ to the heritability of the trait, $P_{HET}$ to the proportion of SNPs with genetic heterogeneity, $\beta_{anc}$ to the effect size in the corresponding ancestry, and $P_{EUR}$ to the proportion of European-ancestry individuals.

We simulate data under a wide range of scenarios following the procedure represented in Figure 1 and further described in the supplemental methods. In brief, we extract common variants from the 1000 Genomes project^27^ by filtering out sites with a minor allele frequency lower than 1% in all ancestries. In each simulation, we sample a given number of variants from these and genotypes are simulated at the subpopulation level with the corresponding allelic frequencies under Hardy-Weinberg equilibrium. We randomly choose four subpopulations in each of the five 1000 Genomes ancestry groups: African (GWD, LWK, MSL, YRI), American (CLM, MXL, PEL, PUR), East-Asian (CDX, CHB, JPT, KHV), European (FIN, GBS, TSI, IBS), and South-Asian (BEB, GIH, PJL, STU). We specify a varying proportion of EUR individuals, with the four remaining non-EUR populations being of equal sample size. We then simulate phenotypes as y=βG+ε, where G corresponds to the simulated genotypes and $\varepsilon ∼N\left(0,\sqrt{1-h_{SNP}^{2}}\right)$, $h_{SNP}^{2}$ being the heritability of the trait. The β vector corresponds to the effect sizes of the variants generated through the log-normal model and further adjusted on $h_{SNP}^{2}$, with all the simulated variants being considered as causal. The log-normal model has been shown to be well-adapted to model common variant effect sizes.^28^ Finally, we specify a proportion of variants with heterogeneous effects across populations. For those, we draw non-null genetic effect sizes in only one ancestry, as opposed to homogeneous variants, for which we draw identical, non-null effects across all of the five ancestries. The same set of simulations are performed twice: once to simulate a base sample where we perform GWASs to estimate variant effect sizes and select the significant variants for PGS construction and once to simulate a target sample on which we apply PGSs and correlate them with the simulated phenotypes. Unless otherwise stated, 150,000 individuals are simulated both in the base and in the target sample. We consider the four PGSs aforementioned: AS, FETA, RETA, and TAMR. Only SNPs meeting the genome-wide significance threshold (P < 5 × 10^−8^) in the AS, FETA, RETA, and TAMR analyses are included in the corresponding PGS. We use R-squared values to assess the predictive performance of the PGS under a direct model where we consider that individual data are available or under an indirect model relying on summary statistics. We report results in each of the five 1000 Genomes ancestries by averaging the measures of fit across the corresponding subpopulations.

**Figure 2:**
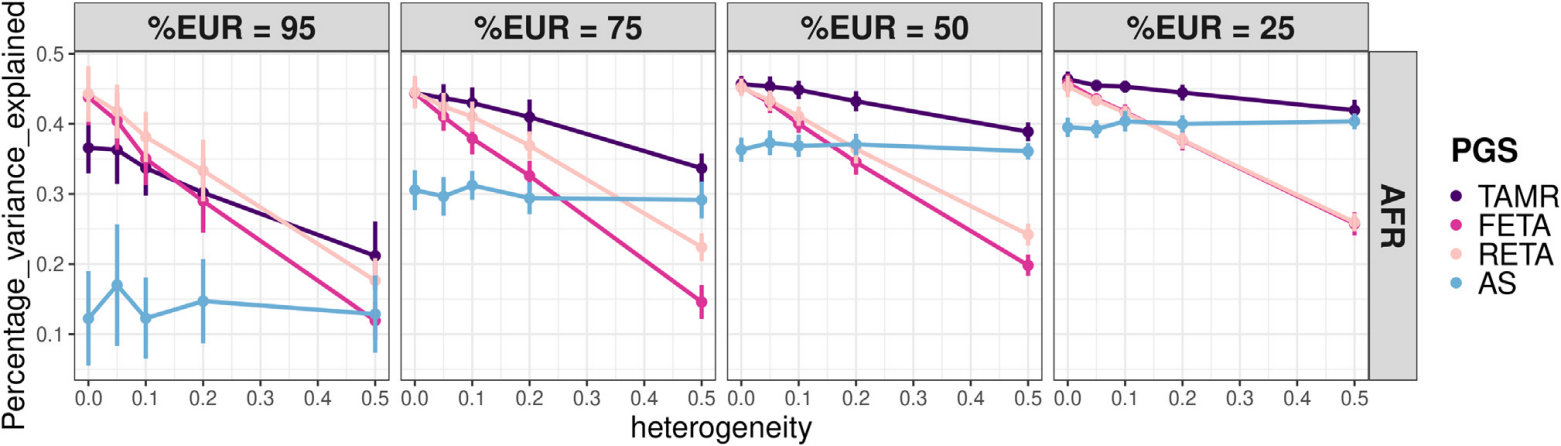
Assessment of the impact of the simulated heterogeneity on the accuracy of the four PGSs TAMR, FETA, RETA, and AS 500 causal SNPs are simulated with a heritability of 50%. PGS performances are evaluated in the African population from the 1000 Genomes project for four percentages of European-ancestry individuals using the direct method. Error bars represent the standard error of the mean variance explained across 10 simulation replicates.

Overall, varying the heritability or the number of causal SNPs in the simulations affects the absolute prediction performance of the three PGSs but does not impact the relative performance between them (Figures S1 and S2). This is in concordance with previous results from Wang et al. who showed that the number of SNPs and extent of heritability do not strongly impact the percentage of variance retrieved from the original simulated value.^14^ Similar prediction performance is found for all of the non-EUR populations and, as expected, the direct method leads to higher prediction levels than the indirect method. We therefore focus on the results observed in the African population using the direct method. The ancestry-related heterogeneity is the most impactful parameter and relative predictions of the four PGSs further depend on the percentage of EUR individuals in the base dataset under simulations (Figure 2). AS PGS is insensitive to the percentage of heterogeneity, as it is constructed in only one population. When no heterogeneity is present, all meta-analysis methods present an advantage over the AS PGS. All methods except AS are negatively impacted by increasing proportions of heterogeneity, to a greater extent for the FETA method, which loses close to 25% of performance when increasing heterogeneity from 0% to 50% when half of the sample is composed of EUR individuals. In comparison, the performance loss is lower than 10% for TAMR. As expected, RETA PGS offers an advantage over FETA PGS when genetic heterogeneity is present but this advantage decreases with an increasing proportion of non-EUR. RETA still remains negatively impacted by an increasing genetic heterogeneity, leading to poorer prediction performance than TAMR PGS in ancestry-diverse samples. The performance of all scores in AFR improves as the proportion of non-EUR in the base sample increases. Notably, AS outperforms FETA after a given level of heterogeneity. This threshold lowers as the proportion of non-EUR increases, which makes AS a better choice than FETA, and even RETA, in diverse samples, even if the suspected heterogeneity is low (20%–30%). While TAMR is also mildly impacted by heterogeneity, it maintains the best performance in most of the situations.

**Figure 3:**
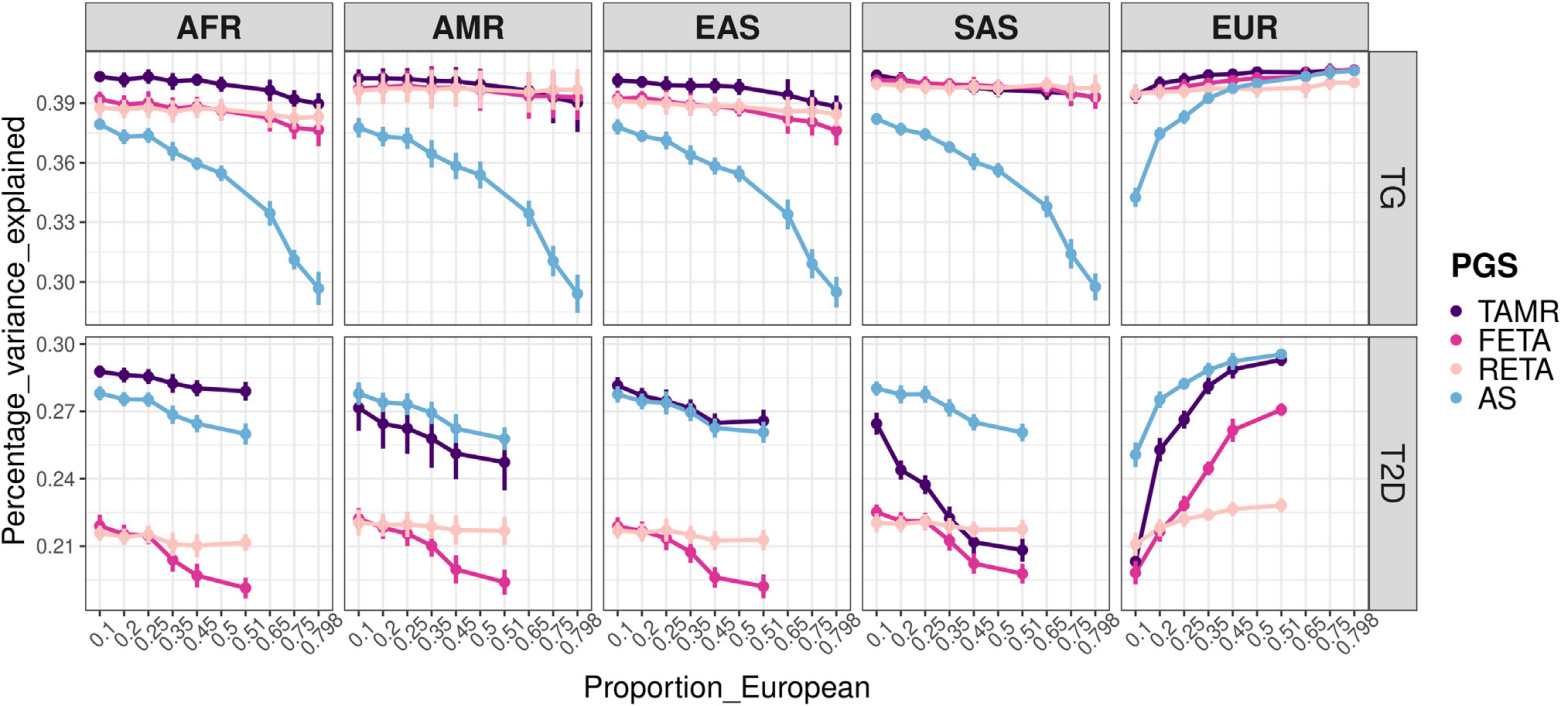
Evaluation of the four PGS constructions for two human complex traits, triglycerides and type 2 diabetes, in the five 1000 Genomes populations Non-European populations are simulated in equal sample sizes. Error bars represent the standard error of the mean variance explained across 10 simulation replicates.

Our comparisons show that AS and FETA/RETA PGS are the most strongly impacted by the sample size and the ancestry-related genetic heterogeneity, respectively. We next perform simulations under real-world scenarios for two complex traits that vary in heterogeneity, heritability and polygenicity, and have been recently investigated in large trans-ancestry studies: T2D^22^ for which we simulate underlying liability, and triglyceride levels^29^ (TGs). Both traits were recently studied in large trans-ancestry meta-analyses gathering more than 180,000 cases and 1.1 million controls with 48.9% non-EUR samples for T2D and more than 1.65 million individuals with 20.2% non-EUR samples for TGs. Since our objective is to evaluate the ideal ancestry composition of future GWASs, we assume that these simulations capture the full genetic architecture of the traits (details are given in the supplemental methods). Briefly, we extrapolate the results from these two studies to inform the simulation parameters described in Table S1. We use MR-MEGA to estimate genetic heterogeneity of effect sizes in the two studies, which yielded a much higher value for T2D (30%) than for TGs (1%). The heritability is estimated from previous family-based studies as 0.42 for TGs^30^ and 0.31 for T2D,^31^ and considered to be the same across ancestral populations. We simulate data by gradually decreasing the proportion of EUR individuals, starting with the level present in those studies’ samples, to demonstrate the benefits of increasing population diversity in GWASs. For TGs, the non-EUR AS PGS increases in prediction performance with decreasing European-ancestry proportion but never reaches the prediction levels of the three PGSs based on trans-ancestry meta-analyses, in concordance with previous scenarios of low heterogeneity (Figure 3 – top panel). TAMR, FETA, and RETA PGS show high prediction levels, stable across the percentage of European-ancestry individuals, probably due to the low heterogeneity simulated. There is still a substantial increase in accuracy in all non-EUR populations when they represent at least 50% of the overall sample. For the AS PGS, optimal predictions in all populations are reached when 20% of EUR samples are present, i.e., when all the populations contribute equally to the meta-analysis, predictions in EUR being dramatically lower when decreasing this percentage. In contrast, for T2D, FETA PGS has a poorer performance than TAMR, RETA, and AS PGS, in line with the results from Figure 2 with a heterogeneity of 30%. Different patterns are observed between African/East-Asian and American/South-Asian populations for AS, RETA, and TAMR. In African and East-Asian populations, AS and TAMR methods show comparable predictive performance that are also higher than RETA. In American and South-Asian populations, there is a loss of accuracy of the TAMR method compared with the AS score and even compared with the RETA score when a high proportion of EUR is simulated. As the African and East-Asian populations are genetically more distinct than the American and South-Asian populations (Figure S3), we hypothesize that the advantage of TAMR over AS PGS in these two populations is because it better models the ancestry-related heterogeneity. Differences in South-Asian and American populations could also be due to other factors not captured by the ancestry-related heterogeneity determined from PCA. This is supported by the fact that RETA PGS, which models heterogeneity but not as a function of genetic distance, is better than TAMR in the South-Asian population. This is also in concordance with a recent study from Wang et al. showing that differences in allelic frequencies and LD patterns between populations have less impact on PGS accuracy in the South-Asian population compared with East-Asian and African populations.^14^ In addition, Huang et al. showed that there is a large overlap of cardiometabolic loci between South-Asian and EUR populations.^32^ For TAMR, AS, and FETA, the optimal mean performance for T2D across populations is observed when the sample is ancestry-balanced, with modest increases in predictions in non-EUR populations when less than 20% of EUR samples are simulated but a large decrease in predictions in EUR. In this optimal setting and even with a higher proportion of non-EUR samples, accuracy levels currently observed in the EUR population are difficult to reach in non-EUR populations. This is especially true in American and South-Asian populations where there is a lower benefit of the TAMR PGS. To verify whether the same trends were observed for other trans-ancestry PGSs, we applied the recently developed CT-SLEB method. CT-SLEB is based on a clumping and threshold approach where multiple pruning parameters and p value thresholds are used to construct PGSs that are then combined using a super-learning algorithm.^33^ The impact of the ancestry composition on the predictions depends on the genetic heterogeneity underlying the trait (Figure S4). When there is low heterogeneity, CT-SLEB performance decreases with the percentage of EUR, while the inverse trend is observed in T2D where a higher heterogeneity is simulated. As the sample size of the discovery set in the EUR and in the target population are important in CT-SLEB, we hypothesize that lower predictions are observed when a lower proportion of EUR is simulated as the sample size decreases, while this is compensated for by the additional information brought by non-EUR samples when there is at least genetic heterogeneity. Overall, we confirm that there is not one method with the best predictions in all scenarios but confirm the advantage of trans-ancestry PGS in non-EUR populations from ancestry-diverse samples, including TAMR and CT-SLEB, using scenarios approximating the genetic architecture of these two traits. This highlights the relevance of these methods for compensating current bias in GWASs linked to unbalanced ancestry compositions.

Overall, genetic heterogeneity and its relationship with ancestry composition of the discovery sample have the greatest impact on the relative accuracy of the methods. This was observed both on theoretical scenarios and on scenarios approaching real human complex traits, T2D and TGs. Our results highlight the advantage of meta-analysis approaches that model ancestry-related genetic heterogeneity, such as meta-regression, which offers optimal accuracy levels. Nevertheless, even this method benefits from the inclusion of non-EUR individuals, especially if there is genetic heterogeneity underlying the trait of interest, emphasizing the need to include more diverse samples in future studies.

Our simulations were based on several simplifying assumptions to reduce computational burden. First, our simulations are based on independent variants to mimic a classical approach where PGS is built upon variants obtained from clumping and selection based on the p value of association.^34^ We did not study the impact of LD, but we hypothesize that while it can have an impact on the absolute power of the methods, it will not modify the relative performance of the PGSs included in this study, as they were all built up only on the significant SNPs. We acknowledge the fact that many other PGS constructions exist, especially integrating all variants in the genome, but this comparison is beyond the scope of this work, which focuses on comparing the accuracy of meta-analysis methods. It is possible that genome-wide PGSs could have a better transferability to non-European populations, but we argue that the conclusions would remain similar in supporting the need to increase diversity at the study design in GWASs. Second, SNPs are all simulated as associated with the phenotype and we did not assess the impact of adding non-causal variants for computational reasons. The simulation of non-causal variants could result in lower performance compared with our simulations even if only a small fraction of these variants is expected to pass the significance threshold required for inclusion in the PGS. Third, only common variants have been considered in the traps simulation framework. Rare variants have also been shown to add predictive value of human complex traits^35^ and tend to be most heterogeneously distributed among ancestries than common variants. The transferability of PGS to non-European populations in our simulations is therefore likely to be over-estimated, especially in the African populations. Fourth, for real trait scenarios, we estimate the parameters under the hypothesis that the full genetic architecture of TGs and T2D is known. Estimating the heterogeneity parameter is particularly hard, because the power to detect heterogeneous variants is itself limited by non-EUR sample size. It is therefore possible that the simulated heterogeneity is underestimated, which could lead to an overestimation of the performance in our simulations. Fifth, in our real-life scenarios, we extrapolate the number of additional causal SNPs required to explain the full narrow-sense heritability of the trait. We assumed this number grows linearly with heritability explained; however, we expect to identify variants with smaller effect sizes explaining a lower proportion of the phenotypic variance as sample sizes increase in future EUR-based GWASs. In non-EUR populations, Morales et al. showed that a higher number of genetic associations is also expected to emerge in future genetic studies from the inclusion of African and Hispanic populations due to their higher genetic diversity and lower LD.^36^ Overall, while our simulation parameters may suffer from biases, we argue that although these biases might lead to an overestimation of the expected overall performance, they are not likely to impact the relative performance of the three PGS methods and the overall conclusion of this work. Moreover, all these limits highlight the difficulty in accurately estimating PGS predictions expected for a wide range of human complex traits, which highly depend on the underlying genetic architecture, and especially the heterogeneity of the trait. Finally, we assumed PGSs were transferrable at the population level. However, even within an ancestry-matched group, PGS predictions can be affected by sample variables such as the age or the sex of individuals,^37^ or even at fine-scale within a population.^38^ Our simulations are conducted using the five 1000 Genomes populations, but considering subpopulations would probably be needed to perform a more comprehensive evaluation. Veturi et al., for example, highlighted heterogeneity in effect sizes between European-Americans and African-Americans^39^ and Kamiza et al. showed that PGS predictions greatly varied between the sub-Saharan and the Ugandan populations in Africa.^40^ This assessment is limited by the use of the 1000 Genomes project itself for the simulations, as studies have shown that most subpopulations in the project actually represent the same underlying ancestry, especially in the SAS population.^41^

In summary, our results from simulations both on hypothetical and real trait scenarios show that PGS accuracy highly depends on the underlying genetic architecture of the trait, especially heterogeneity among populations. In traits with higher levels of genetic heterogeneity, TAMR PGS offers advantages compared with a fixed-effects meta-analysis or an AS score but the performances are still low given the current ancestry distribution. Predictions in non-European populations in this setting will also most probably benefit from the recent developments in trans-ancestry PGS given the results obtained with CT-SLEB in T2D scenarios. Our simulation results are in line with other studies highlighting the poor transferability of PGS to non-EUR populations^11^^,^^14^ and support the urgent need to increase diversity in future genetic studies. Several large genetic projects are moving in that direction, such as the Uganda Genome Resource,^42^ the H3Africa project,^43^^,^^44^ or the GenomeAsia 100K project^45^ focusing on specific large ancestries, and the PAGE study^10^ or the IHCC^46^ integrating multi-continental ancestries. This translation will require new collaborations and a shift in the overall conception of genetic studies^5^^,^^47^ but will undoubtedly lead to better comprehension of human complex traits. The translation to non-EUR populations represents the next step to widely accessible precision medicine^48^ and is crucial to redress existing health disparities where using European-based data could lead to wrong conclusions in other populations.^13^

## Data Availability

The traps procedure is available at https://github.com/hmgu-itg/traps along with examples.

## Supplemental information

Document S1. Figures S1–S4, Table S1, and supplemental methods

Document S2. Article plus supplemental information
