# Supplementary material for: Bridging the diversity gap: Analytical and study design considerations for improving the accuracy of trans-ancestry genetic prediction
Source: HGG Adv. 2023 Jun 15;4(3):100214. doi: 10.1016/j.xhgg.2023.100214 (PMC10336686; doi:10.1016/j.xhgg.2023.100214)
Supplement: Document S1. Figures S1–S4, Table S1, and supplemental methods [file mmc1.pdf]

HGGA, Volume *4*

Supplemental information

Bridging the diversity gap: Analytical and study design considerations for improving the accuracy of trans-ancestry genetic prediction

Ozvan Bocher, Arthur Gilly, Young-Chan Park, Eleftheria Zeggini, and Andrew P. Morris

Supplemental Figures

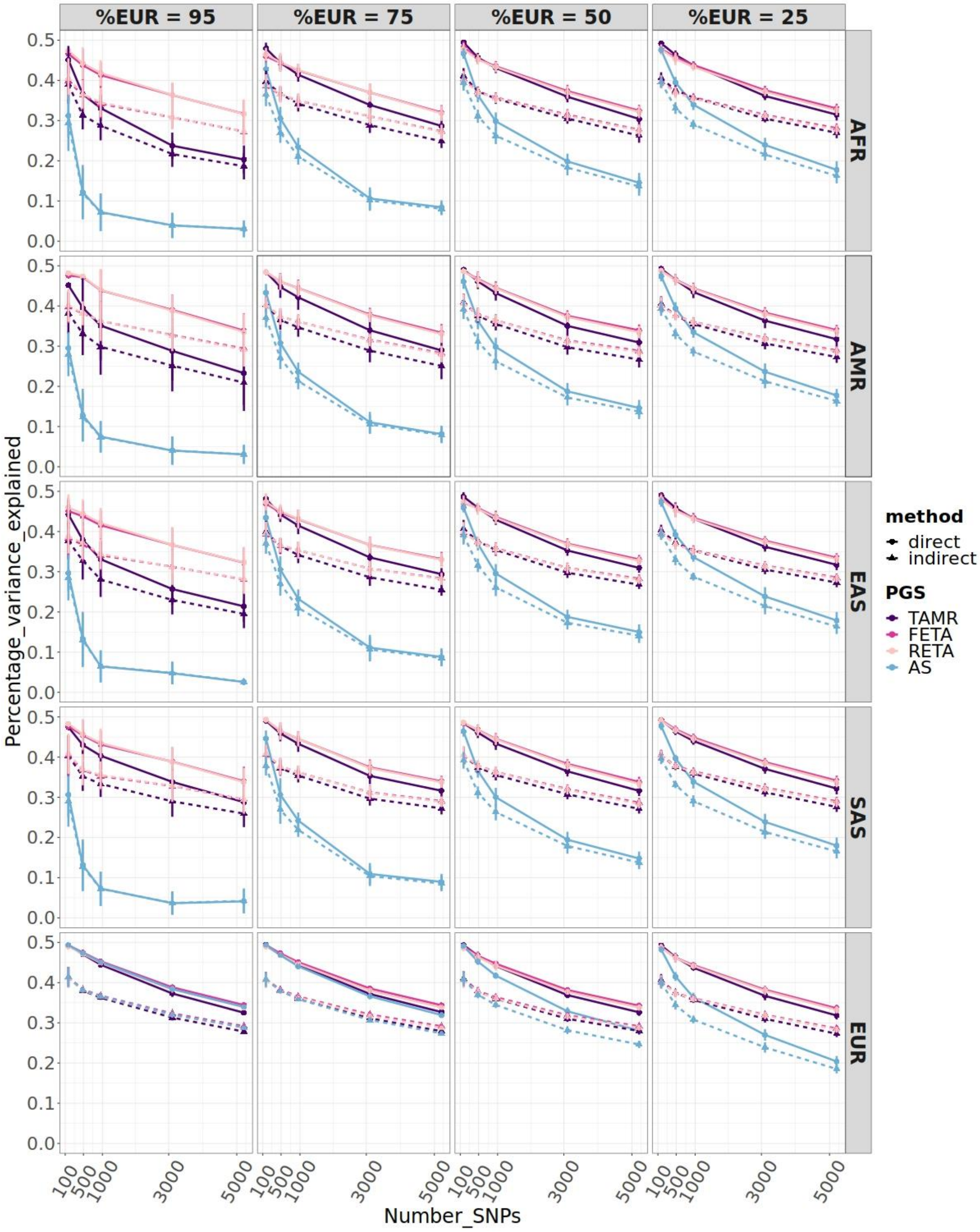

Figure S1: Assessment of the impact of the number of causal SNPs on the accuracy of the three PGS TAMR, FETA and AS. A heritability of 50% was simulated with no heterogeneity. PGS accuracies were evaluated in the five 1000 Genomes populations for four percentages of European-ancestry individuals. Error bars represent the standard error of the mean variance explained across 10 simulation replicates.

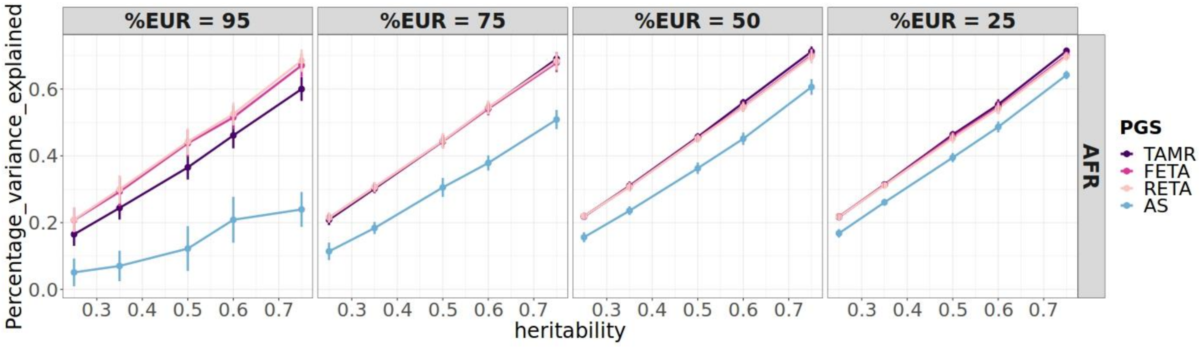

Figure S2: Assessment of the impact of the simulated heritability on the accuracy of the three PGS TAMR, FETA and AS. 500 causal SNPs were simulated with no heterogeneity. PGS accuracies were evaluated in the African population from the 1000 Genome project for four percentages of European-ancestry individuals. Error bars represent the standard error of the mean variance explained across 10 simulation replicates.

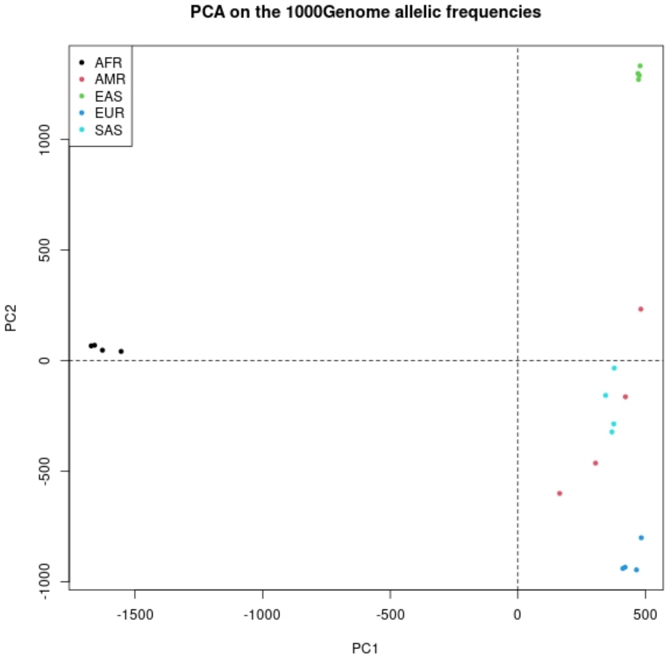

Figure S3: PCA on the allelic frequencies from 20 subpopulations from the 1000Genomes project used for the simulations

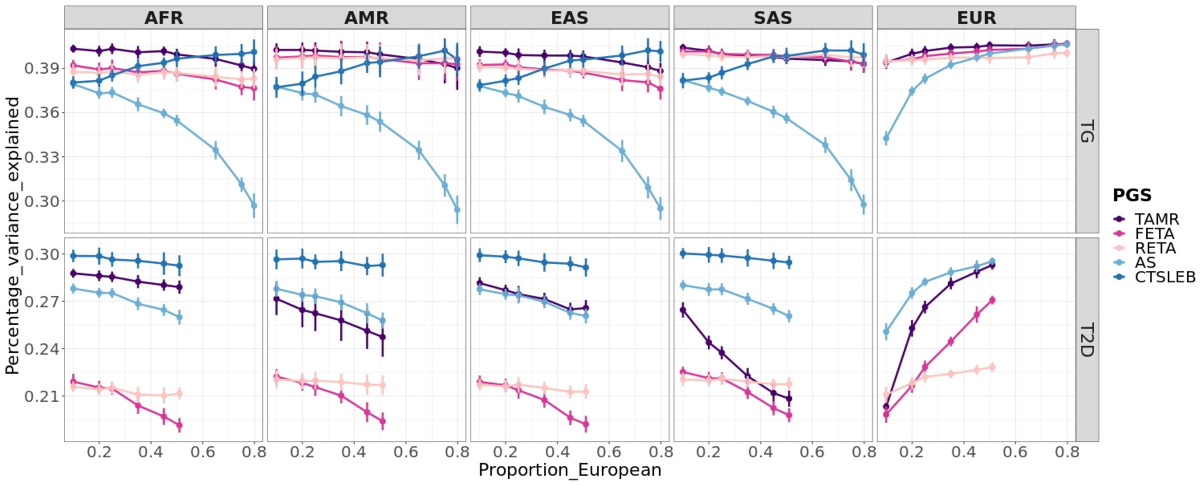

Supplementary Figure 4: Evaluation of five PGS constructions for two human complex traits, Triglycerides and Type 2 Diabetes, in the five 1000Genomes populations. Non-European populations are simulated in equal sample sizes. Error bars represent the standard error of the mean variance explained across 10 simulation replicates. This figure corresponds to Figure 3 in the main text with the additional method CT-SLEB being tested.

Supplemental tables

		TG	T2D
**Original study**	Study	10.1038/s41586-021-04064-3	10.1038/s41588-022-01058-3
	Number of independent SNP	461	338
	Variance explained (%)	9	8
	Sample size	1,654,960	1,339,889
	EUR/AFR/AMR/EAS/SAS (%)	79.8/6/2.9/8.8/2.5	51.1/6.6/5.6/28.4/8.3
	Heterogeneity (%)	19	40
**Simulations**	Sample size	1,654,960	1,339,889
	EUR/AFR/AMR/EAS/SAS (%)	79.8/5.3 (x4)<br>75/6.25 (x4)<br>65/8.75 (x4)<br>50/12.5 (x4)<br>45/13.75 (x4)<br>35/16.25 (x4)<br>25/18.75 (x4)<br>20 (x5)<br>10/22.5 (x4)	51.1/12.225 (x4)<br>45/13.75 (x4)<br>35/16.25 (x4)<br>25/18.75 (x4)<br>20 (x5)<br>10/22.5 (x4)
	Heterogeneity (%)	1	30
	Heritability (%)	42	31
	Number of independent SNP	2151	1310

Table S1: Parameters estimated from two studies on TGs and T2D and the corresponding values in the traps simulations.

Supplemental methods

Overview of the pipeline

We have developed a pipeline for TRans Ancestry PGS Simulation (traps, https://github.com/hmgu-itg/traps) that simulates genetic data using 1000Genomes project allelic frequencies[1], performs GWAS, constructs PGS and assesses their performance in multiple ancestry groups. We randomly choose four subpopulations in each of the five 1000Genomes population groups: AFR (GWD, LWK, MSL, YRI), AMR (CLM, MXL, PEL, PUR), EAS (CDX, CHB, JPT, KHV), EUR (FIN, GBS, TSI, IBS) and SAS (BEB, GIH, PJL, STU). We then simulate genetic data and perform association tests within each of the 20 subpopulations. The different steps of traps are detailed hereafter.

Simulations

First, we simulate genetic data for a user-specified number of genetic variants sampled from the common variants observed in the 1000Genomes project (overall minor allele frequency or 'MAF' higher than 1%). Genotypes are simulated for individuals in each subpopulation under the Hardy-Weinberg equilibrium using the allelic frequencies observed in the corresponding 1000Genomes subpopulation (drawn genetic variants are considered independent). The sample's ancestry composition, expressed either as a proportion of Europeans with the remaining population equally distributed, or five ancestry-specific proportions summing to one, is specified by the user. Unless otherwise stated, 150,000 individuals are simulated in this work, and the first method is used to specify ancestry composition.

Second, we simulate an associated phenotype by drawing a genetic effect w at random for each variant (note that all simulated variants are considered as causal) according to the log-normal model[2] with $log10(w) \sim N(-log10(M) - 0.8^2, 0.8)$, M representing the total number of causal variants.

These effects are then normalized into units of phenotype standard deviation per unit of genotype standard deviation, and further adjusted so that the sum of squared effect sizes is equal to a user-specified heritability value (h^2_{SNP}) as: $\beta_s = \sqrt{\frac{h^2}{\sum_{s=1}^{S} w_s}}$. Finally, continuous phenotypes are simulated as: $y = \beta G + \varepsilon$ where β are the normalized effect sizes of the SNPs, G the corresponding genotypes and $\varepsilon \sim N\left(0, \sqrt{1 - h^2_{SNP}}\right)$.

We apply this simulation procedure once for generating a "base" dataset used for PGS construction, and once for a "target" dataset used in PGS evaluation.

Association analysis

After simulating genotypes and phenotypes, we perform association tests in each of the 20 subpopulations in the base and in the target samples separately using the PLINK software[3]. Three meta-analyses are then performed to obtain SNPs weights for combined PGS:

1. Fixed-Effect Trans-Ancestry (FETA) which is a classical fixed-effect cross-ancestry meta-analysis across all subpopulations using the METASOFT software[4].
2. Random-Effect Trans-Ancestry (RETA) which is a classical random-effect cross-ancestry meta-analysis across all subpopulations using the RE2 model from the METASOFT software[4]
3. Trans-Ancestry Meta-Regression (TAMR) which applies the meta-regression from Magi et al.[5] where axes of genetic variation representing the ancestry of the individuals model the heterogeneity related to ancestry between the effects in the different subpopulations. These axes of genetic variation are integrated along with estimates from GWAS in the base sample

to produce one adjusted effect per subpopulation. In traps, we use the first three principal components of the PCA on the 1000Genomes project allelic frequencies for this adjustment.

PGS construction and evaluation

SNP effect sizes from the meta-analysis serve as weights for the corresponding PGS which are then applied to the 20 target subpopulations. For FETA and RETA, the same effects from the trans-ancestry meta-analysis are applied to all samples irrespective of their origin. For TAMR, ancestry-adjusted effects computed in each subpopulation are used to compute PGS. We select SNPs with a p-value lower than 5x10^{-8} in the base sample to mimic commonly used PGS approaches[6]. In addition to the two meta-analysis PGS, an ancestry-specific (AS) PGS is computed in every population using the same significance threshold and corresponding SNP weights from GWAS in the base sample.

We evaluate the three PGS using either a 'direct' or an 'indirect' method. In the direct method, we suppose that individual data are available and PGS are computed for each individual j as: $PGS_j = \sum_{s=1}^{S} \beta_s G_{j,s}$. For quantitative traits, PGS_j is the predicted phenotype, while for binary traits, it is the predicted genetic liability. R^2 is used as a measure of fit. When only summary statistics are available, goodness of fit can be measured indirectly by:

$$1 - \exp\left(\left[\frac{\sum_{s=1}^{S} \beta_{b,s} \times \beta_{t,s} \times sd_{t,s}^{-2}}{\sum_{s=1}^{S} \beta_{t,s} \times sd_{t,s}^{\ -2}} \times \frac{1}{\sqrt{\frac{1}{\beta_{t,s}^2 \times sd_{t,s}^{\ -2}}}}\right]^2 \times \frac{1}{n}\right)$$

With β_b and β_t the estimated effects in the base and target samples respectively, sd_t the corresponding standard deviation and n the sample size of the target sample. More details can be found in Dastani et al.[7] and have been implemented in the gtx R package[8]. Finally, we average R^2 across subpopulations to produce one measure of fit per ancestry group.

Simulated scenarios

General comparisons of parameters

We simulate 56 scenarios corresponding to a grid where we varied the following parameters: (1) the number of SNPs sampled from the 1000Genomes allele frequencies; (2) the heritability of the trait on which SNP effects are adjusted; (3) the proportion of European-ancestry individuals; (4) the percentage of SNPs with population-specific genetic effects. For each scenario, we perform 10 replicates and average accuracy.

Scenarios approximating real traits

Second, we simulate data under real-world scenarios for two complex traits that have been recently investigated in large trans-ancestry studies: type 2 diabetes[9] (T2D) for which we simulate underlying liability, and triglycerides levels[10] (TGs). Since our objective is to evaluate the ideal ancestry composition of future GWAS, we assume that these simulations capture the full genetic architecture of the traits. We estimate the expected number of associated SNPs as: $M = M_d * \frac{h^2}{h_d^2}$ with M_d corresponding to the number of independent SNPs associated at genome-wide significance in these two studies, h_d^2 the cumulative percentage of variance they explain and h^2 the heritability of the trait estimated from family-based studies. The heritability was estimated in previous studies as 0.42 for TGs[11] and 0.31 for T2D[12]. The proportion of heterogeneity was estimated in each study as the proportion of SNPs having a nominally significant p-value of heterogeneity due to ancestry, as

provided by MR-MEGA. We selected the proportion of heterogeneous SNPs in our simulations such that, across simulated data sets, the estimated proportion of SNPs with nominal evidence ($P<0.05$) of ancestry-correlated heterogeneity matched that observed in the real data. Finally, we assess PGS predictions using increasingly diverse sample from the original studies. We present figures from the two studies and the corresponding parameter values for the simulations in Table S1.

Supplemental references

1 Sudmant, P. H. *et al.* An integrated map of structural variation in 2,504 human genomes. *Nature* **526**, 75-81 (2015). https://doi.org:10.1038/nature15394

2 O'Connor, L. J. The distribution of common-variant effect sizes. *Nat Genet* **53**, 1243-1249 (2021). https://doi.org:10.1038/s41588-021-00901-3

3 Purcell, S. *et al.* PLINK: a tool set for whole-genome association and population-based linkage analyses. *Am J Hum Genet* **81**, 559-575 (2007). https://doi.org:10.1086/519795

4 Han, B. & Eskin, E. Random-effects model aimed at discovering associations in meta-analysis of genome-wide association studies. *Am J Hum Genet* **88**, 586-598 (2011). https://doi.org:10.1016/j.ajhg.2011.04.014

5 Magi, R. *et al.* Trans-ethnic meta-regression of genome-wide association studies accounting for ancestry increases power for discovery and improves fine-mapping resolution. *Hum Mol Genet* **26**, 3639-3650 (2017). https://doi.org:10.1093/hmg/ddx280

6 Choi, S. W., Mak, T. S. & O'Reilly, P. F. Tutorial: a guide to performing polygenic risk score analyses. *Nat Protoc* **15**, 2759-2772 (2020). https://doi.org:10.1038/s41596-020-0353-1

7 Dastani, Z. *et al.* Novel loci for adiponectin levels and their influence on type 2 diabetes and metabolic traits: a multi-ethnic meta-analysis of 45,891 individuals. *PLoS Genet* **8**, e1002607 (2012). https://doi.org:10.1371/journal.pgen.1002607

8 gtx (R CRAN, 2019).

9 Mahajan, A. *et al.* Multi-ancestry genetic study of type 2 diabetes highlights the power of diverse populations for discovery and translation. *Nat Genet* **54**, 560-572 (2022). https://doi.org:10.1038/s41588-022-01058-3

10 Graham, S. E. *et al.* The power of genetic diversity in genome-wide association studies of lipids. *Nature* **600**, 675-679 (2021). https://doi.org:10.1038/s41586-021-04064-3

11 Blackburn, N. B., Porto, A., Peralta, J. M. & Blangero, J. Heritability and genetic associations of triglyceride and HDL-C levels using pedigree-based and empirical kinships. *BMC Proc* **12**, 34 (2018). https://doi.org:10.1186/s12919-018-0133-x

12 Almgren, P. *et al.* Heritability and familiality of type 2 diabetes and related quantitative traits in the Botnia Study. *Diabetologia* **54**, 2811-2819 (2011). https://doi.org:10.1007/s00125-011-2267-5
