# Supplementary material for: Bridging the diversity gap: Analytical and study design considerations for improving the accuracy of trans-ancestry genetic prediction
Source: HGG Adv. 2023 Jun 15;4(3):100214. doi: 10.1016/j.xhgg.2023.100214 (PMC10336686; doi:10.1016/j.xhgg.2023.100214)
Supplement: Document S2. Article plus supplemental information [file mmc2.pdf]

HGG
Advances

REPORT

Bridging the diversity gap: Analytical and study design considerations for improving the accuracy of trans-ancestry genetic prediction

Ozvan Bocher,[1,5,*] Arthur Gilly,[1] Young-Chan Park,[1] Eleftheria Zeggini,[1,2,3] and Andrew P. Morris[1,4]

Summary

Genetic prediction of common complex disease risk is an essential component of precision medicine. Currently, genome-wide association studies (GWASs) are mostly composed of European-ancestry samples and resulting polygenic scores (PGSs) have been shown to poorly transfer to other ancestries partly due to heterogeneity of allelic effects between populations. Fixed-effects (FETA) and random-effects (RETA) trans-ancestry meta-analyses do not model such ancestry-related heterogeneity, while ancestry-specific (AS) scores may suffer from low power due to low sample sizes. In contrast, trans-ancestry meta-regression (TAMR) builds ancestry-aware PGS that account for more complex trans-ancestry architectures. Here, we examine the predictive performance of these four PGSs under multiple genetic architectures and ancestry configurations. We show that the predictive performance of FETA and RETA is strongly affected by cross-ancestry genetic heterogeneity, while AS PGS performance decreases in under-represented target populations. TAMR PGS is also impacted by heterogeneity but maintains good prediction performance in most situations, especially in ancestry-diverse scenarios. In simulations of human complex traits, TAMR scores currently explain 25% more phenotypic variance than AS in triglyceride levels and 33% more phenotypic variance than FETA in type 2 diabetes in most non-European populations. Importantly, a high proportion of non-European-ancestry individuals is needed to reach prediction levels that are comparable in those populations to the one observed in European-ancestry studies. Our results highlight the need to rebalance the ancestral composition of GWAS to enable accurate prediction in non-European-ancestry groups, and demonstrate the relevance of meta-regression approaches for compensating some of the current population biases in GWAS.

Genome-wide association studies (GWASs) have greatly improved our understanding of the genetics of human complex traits, with over 415,000 genetic associations described across approximately 5,900 studies to date.[1] This achievement has been made possible partly through the development of large genotyping and sequencing projects including the UK Biobank,[2] the TOPMed program,[3] and the FinnGen study.[4] Although GWASs have reached unprecedented sample sizes, European-ancestry (EUR) individuals still dominate their ancestry composition. They commonly represent around 80% of sample sizes, a ratio that has not decreased in the last few years.[5] Human populations differ in linkage disequilibrium patterns, allelic frequencies, and exposures to environmental factors.[6,7] As a result, common variants associated with a wide range of complex phenotypes tend to have different effect sizes between populations.[8–10] Polygenic scores (PGSs) that aim to predict complex human traits by aggregating observed effects across the genome are built from GWASs and are therefore mainly constructed on EUR samples. Due to the heterogeneous genetic architecture between populations, numerous studies have shown that EUR-based PGSs poorly transfer to non-European-ancestry (non-EUR) populations, especially those at greater genetic distance such as those of African ancestry (AFR).[11–15] A recent study even showed that a current schizophrenia PGS is more correlated with the ancestry of the individuals than with the trait itself.[16] GWASs in non-EUR populations tend to be small if they exist at all, which prevents the development of ancestry-specific (AS) PGSs in those populations. To improve predictions in non-EUR populations, studies from diverse ancestries can be combined through the use of meta-analysis. The most used approach is the fixed-effect model that weights the effect size of a study based on the inverse of the variance, with the assumption that all studies measure the same underlying effect for each variant.[17] These methods perform poorly when genetic heterogeneity is present among contributing studies, a situation that is more likely when combining data from diverse ancestries than when aggregating GWASs from the same population background.[8–10] Random-effect models have been developed to address this issue, but they do not assume any pattern in the heterogeneity between studies and tend to have a limited advantage over fixed-effect meta-analyses.[18] Several further methods aim to specifically answer the question of PGS transferability to non-EUR populations, including the meta-regression model.[19–21] In this framework, axes of genetic variation obtained from principal-component analysis (PCA) on the populations are incorporated in the regression model to represent

[1]ITG, Helmholtz Zentrum München, Munich, Germany; [2]Technical University of Munich, Munich, Germany; [3]Klinikum Rechts der Isar, Munich, Germany; [4]Centre for Genetics and Genomics Versus Arthritis, Centre for Musculoskeletal Research, University of Manchester, Manchester, UK
[5]Lead contact
*Correspondence: ozvan.bocher@helmholtz-munich.de
https://doi.org/10.1016/j.xhgg.2023.100214.
© 2023 The Author(s). This is an open access article under the CC BY license (http://creativecommons.org/licenses/by/4.0/).

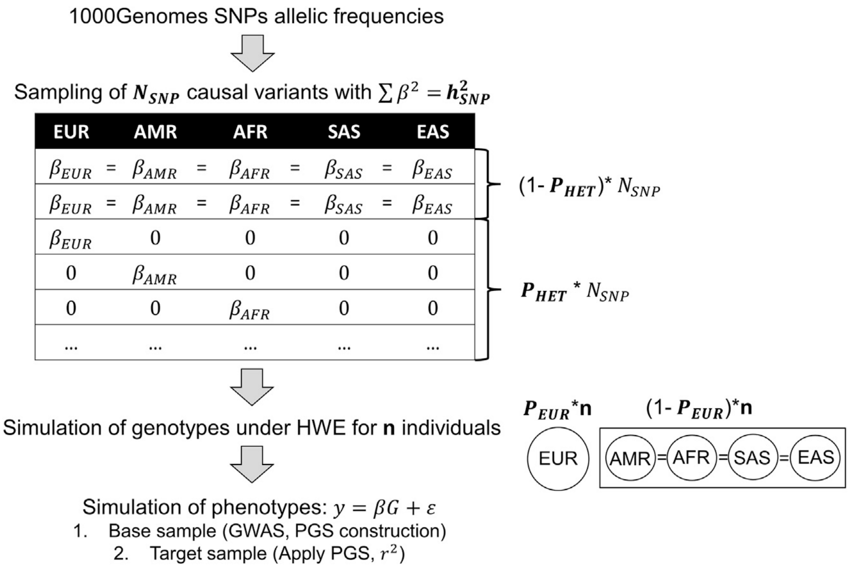

Figure 1. Overview of the traps simulation procedure
N_{SNP} corresponds to the number of causal SNPs simulated, h^2_{SNP} to the heritability of the trait, P_{HET} to the proportion of SNPs with genetic heterogeneity, β_{anc} to the effect size in the corresponding ancestry, and P_{EUR} to the proportion of European-ancestry individuals.

ancestry-related heterogeneity of the genetic effects, leading to a higher predictive power in under-represented populations.[19,22] To date, studies on PGS transferability have mostly focused on evaluating population-specific scores (mainly EUR-based) without extensive evaluation of trans-ancestry PGSs.[11–14,23] Even when trans-ancestry PGSs were evaluated, no extensive assessment considering the genetic architecture of the trait or the impact of the ancestry composition of the sample was performed.[24] This question is important, as trans-ancestry PGSs have been shown to outperform population-specific PGSs for diverse ancestries, including Japanese[25] and Latino.[20] To fill in this gap, we developed a TRans-Ancestry PGS Simulation (traps) procedure to perform a comprehensive assessment of the parameters influencing the transferability of four PGS models in non-EUR populations: an AS PGS (AS), a PGS from a fixed-effect trans-ancestry meta-analysis (FETA), a PGS from a random-effect trans-ancestry meta-analysis (RETA), and PGS from a trans-ancestry meta-regression incorporating ancestry-related heterogeneity (TAMR), implemented in the MR-MEGA software.[19] In this work, we investigate the relative performance of the four approaches via simulation and application to two complex human traits. We chose to focus here on PGS methods constructed from SNPs that meet a predefined significance threshold and therefore did not consider the most recent methods integrating genome-wide SNPs such as PRS-CSx.[24] Indeed, the main purpose of this study was not to perform a comprehensive quantitative comparison of available PGS methods but rather to assess parameters influencing PGS transferability and support the need for trans-ancestry studies. We focused on this class of PGSs as it has been shown to be of similar performance as genome-wide PGSs in type 2 diabetes (T2D), one of our disease models,[26] and because we simulated variants without considering linkage disequilibrium (LD), which is more compatible with PGS built up on significant variants.

We simulate data under a wide range of scenarios following the procedure represented in Figure 1 and further described in the supplemental methods. In brief, we extract common variants from the 1000 Genomes project[27] by filtering out sites with a minor allele frequency lower than 1% in all ancestries. In each simulation, we sample a given number of variants from these and genotypes are simulated at the subpopulation level with the corresponding allelic frequencies under Hardy-Weinberg equilibrium. We randomly choose four subpopulations in each of the five 1000 Genomes ancestry groups: African (GWD, LWK, MSL, YRI), American (CLM, MXL, PEL, PUR), East-Asian (CDX, CHB, JPT, KHV), European (FIN, GBS, TSI, IBS), and South-Asian (BEB, GIH, PJL, STU). We specify a varying proportion of EUR individuals, with the four remaining non-EUR populations being of equal sample size. We then simulate phenotypes as $y = \beta G + \varepsilon$, where G corresponds to the simulated genotypes and $\varepsilon \sim N\left(0, \sqrt{1 - h^2_{SNP}}\right)$, h^2_{SNP} being the heritability of the trait. The β vector corresponds to the effect sizes of the variants generated through the log-normal model and further adjusted on h^2_{SNP}, with all the simulated variants being considered as causal. The log-normal model has been shown to be well-adapted to model common variant effect sizes.[28] Finally, we specify a proportion of variants with heterogeneous effects across populations. For those, we draw non-null genetic effect sizes in only one ancestry, as opposed to homogeneous variants, for which we draw identical, non-null effects across all of the five ancestries. The same set of simulations are performed twice: once to simulate a base sample where we perform GWASs to estimate variant effect sizes and select the significant variants for PGS construction and once to simulate a target sample on which we apply PGSs and correlate them with the simulated phenotypes. Unless otherwise stated, 150,000 individuals are simulated both in the base and in the target sample. We consider the four PGSs aforementioned: AS, FETA, RETA, and TAMR. Only SNPs meeting the genome-wide significance threshold ($P < 5 \times 10^{-8}$) in the AS, FETA, RETA, and TAMR analyses are included in the corresponding PGS. We use R-squared values to assess the predictive performance of the PGS under a direct model where we consider

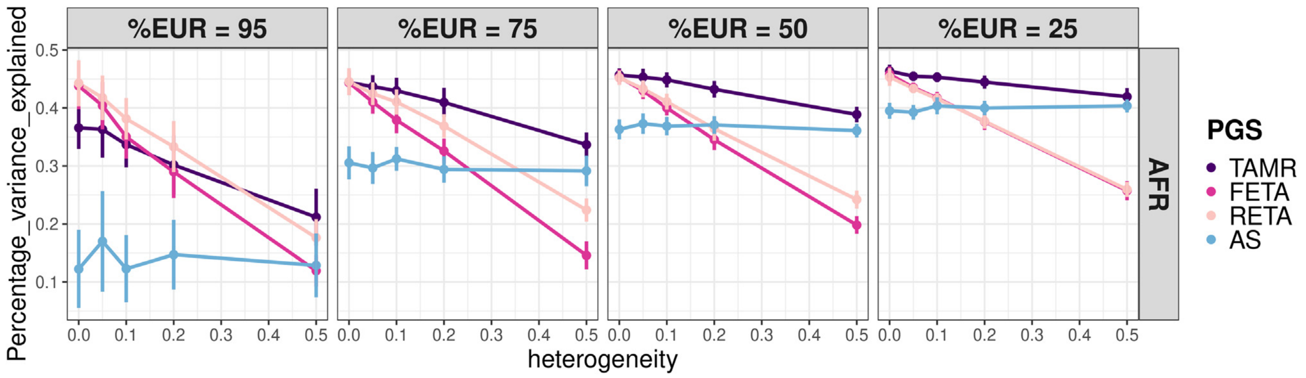

Figure 2. Assessment of the impact of the simulated heterogeneity on the accuracy of the four PGSs TAMR, FETA, RETA, and AS
500 causal SNPs are simulated with a heritability of 50%. PGS performances are evaluated in the African population from the 1000 Genomes project for four percentages of European-ancestry individuals using the direct method. Error bars represent the standard error of the mean variance explained across 10 simulation replicates.

that individual data are available or under an indirect model relying on summary statistics. We report results in each of the five 1000 Genomes ancestries by averaging the measures of fit across the corresponding subpopulations.

Overall, varying the heritability or the number of causal SNPs in the simulations affects the absolute prediction performance of the three PGSs but does not impact the relative performance between them (Figures S1 and S2). This is in concordance with previous results from Wang et al. who showed that the number of SNPs and extent of heritability do not strongly impact the percentage of variance retrieved from the original simulated value.[14] Similar prediction performance is found for all of the non-EUR populations and, as expected, the direct method leads to higher prediction levels than the indirect method. We therefore focus on the results observed in the African population using the direct method. The ancestry-related heterogeneity is the most impactful parameter and relative predictions of the four PGSs further depend on the percentage of EUR individuals in the base dataset under simulations (Figure 2). AS PGS is insensitive to the percentage of heterogeneity, as it is constructed in only one population. When no heterogeneity is present, all meta-analysis methods present an advantage over the AS PGS. All methods except AS are negatively impacted by increasing proportions of heterogeneity, to a greater extent for the FETA method, which loses close to 25% of performance when increasing heterogeneity from 0% to 50% when half of the sample is composed of EUR individuals. In comparison, the performance loss is lower than 10% for TAMR. As expected, RETA PGS offers an advantage over FETA PGS when genetic heterogeneity is present but this advantage decreases with an increasing proportion of non-EUR. RETA still remains negatively impacted by an increasing genetic heterogeneity, leading to poorer prediction performance than TAMR PGS in ancestry-diverse samples. The performance of all scores in AFR improves as the proportion of non-EUR in the base sample increases. Notably, AS outperforms FETA after a given level of heterogeneity. This threshold lowers as the proportion of non-EUR increases, which makes AS a better choice than FETA, and even RETA, in diverse samples, even if the suspected heterogeneity is low (20%–30%). While TAMR is also mildly impacted by heterogeneity, it maintains the best performance in most of the situations.

Our comparisons show that AS and FETA/RETA PGS are the most strongly impacted by the sample size and the ancestry-related genetic heterogeneity, respectively. We next perform simulations under real-world scenarios for two complex traits that vary in heterogeneity, heritability and polygenicity, and have been recently investigated in large trans-ancestry studies: T2D[22] for which we simulate underlying liability, and triglyceride levels[29] (TGs). Both traits were recently studied in large trans-ancestry meta-analyses gathering more than 180,000 cases and 1.1 million controls with 48.9% non-EUR samples for T2D and more than 1.65 million individuals with 20.2% non-EUR samples for TGs. Since our objective is to evaluate the ideal ancestry composition of future GWASs, we assume that these simulations capture the full genetic architecture of the traits (details are given in the supplemental methods). Briefly, we extrapolate the results from these two studies to inform the simulation parameters described in Table S1. We use MR-MEGA to estimate genetic heterogeneity of effect sizes in the two studies, which yielded a much higher value for T2D (30%) than for TGs (1%). The heritability is estimated from previous family-based studies as 0.42 for TGs[30] and 0.31 for T2D,[31] and considered to be the same across ancestral populations. We simulate data by gradually decreasing the proportion of EUR individuals, starting with the level present in those studies' samples, to demonstrate the benefits of increasing population diversity in GWASs. For TGs, the non-EUR AS PGS increases in prediction performance with decreasing European-ancestry proportion but never reaches the prediction levels of the three PGSs based on trans-ancestry meta-analyses, in concordance with previous scenarios of low heterogeneity (Figure 3 – top panel). TAMR, FETA, and RETA PGS show high prediction levels, stable across the percentage of European-ancestry

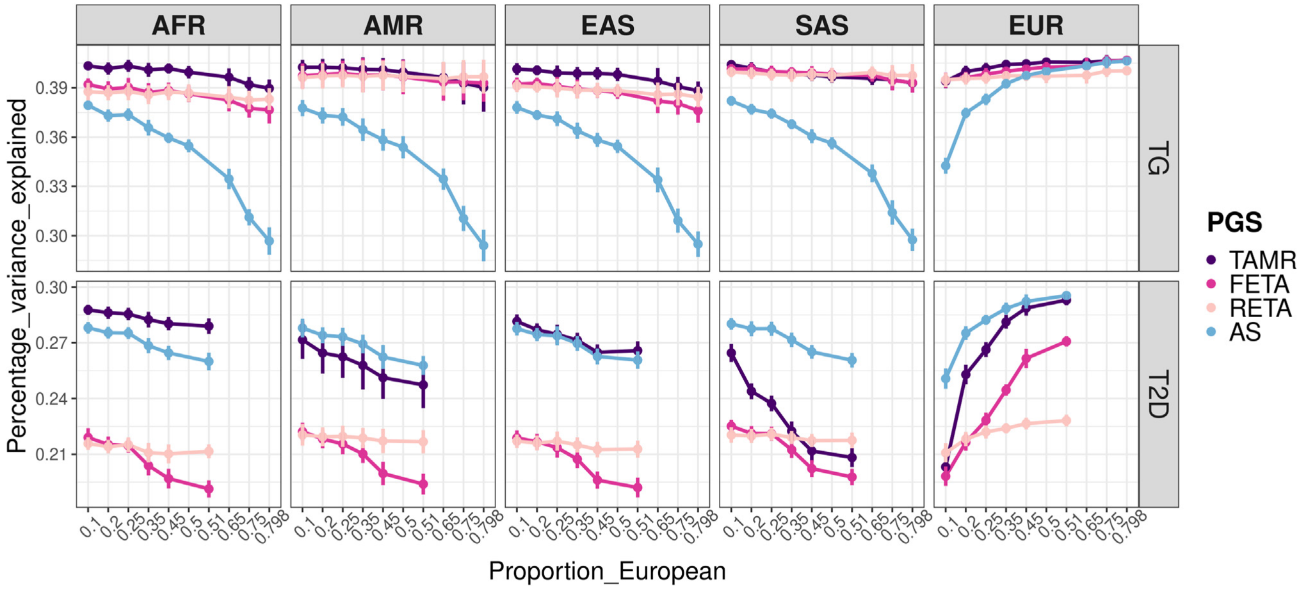

Figure 3. Evaluation of the four PGS constructions for two human complex traits, triglycerides and type 2 diabetes, in the five 1000 Genomes populations
Non-European populations are simulated in equal sample sizes. Error bars represent the standard error of the mean variance explained across 10 simulation replicates.

individuals, probably due to the low heterogeneity simulated. There is still a substantial increase in accuracy in all non-EUR populations when they represent at least 50% of the overall sample. For the AS PGS, optimal predictions in all populations are reached when 20% of EUR samples are present, i.e., when all the populations contribute equally to the meta-analysis, predictions in EUR being dramatically lower when decreasing this percentage. In contrast, for T2D, FETA PGS has a poorer performance than TAMR, RETA, and AS PGS, in line with the results from Figure 2 with a heterogeneity of 30%. Different patterns are observed between African/East-Asian and American/South-Asian populations for AS, RETA, and TAMR. In African and East-Asian populations, AS and TAMR methods show comparable predictive performance that are also higher than RETA. In American and South-Asian populations, there is a loss of accuracy of the TAMR method compared with the AS score and even compared with the RETA score when a high proportion of EUR is simulated. As the African and East-Asian populations are genetically more distinct than the American and South-Asian populations (Figure S3), we hypothesize that the advantage of TAMR over AS PGS in these two populations is because it better models the ancestry-related heterogeneity. Differences in South-Asian and American populations could also be due to other factors not captured by the ancestry-related heterogeneity determined from PCA. This is supported by the fact that RETA PGS, which models heterogeneity but not as a function of genetic distance, is better than TAMR in the South-Asian population. This is also in concordance with a recent study from Wang et al. showing that differences in allelic frequencies and LD patterns between populations have less impact on PGS accuracy in the South-Asian population compared with East-Asian and African populations.[14] In addition, Huang et al. showed that there is a large overlap of cardiometabolic loci between South-Asian and EUR populations.[32] For TAMR, AS, and FETA, the optimal mean performance for T2D across populations is observed when the sample is ancestry-balanced, with modest increases in predictions in non-EUR populations when less than 20% of EUR samples are simulated but a large decrease in predictions in EUR. In this optimal setting and even with a higher proportion of non-EUR samples, accuracy levels currently observed in the EUR population are difficult to reach in non-EUR populations. This is especially true in American and South-Asian populations where there is a lower benefit of the TAMR PGS. To verify whether the same trends were observed for other trans-ancestry PGSs, we applied the recently developed CT-SLEB method. CT-SLEB is based on a clumping and threshold approach where multiple pruning parameters and p value thresholds are used to construct PGSs that are then combined using a super-learning algorithm.[33] The impact of the ancestry composition on the predictions depends on the genetic heterogeneity underlying the trait (Figure S4). When there is low heterogeneity, CT-SLEB performance decreases with the percentage of EUR, while the inverse trend is observed in T2D where a higher heterogeneity is simulated. As the sample size of the discovery set in the EUR and in the target population are important in CT-SLEB, we hypothesize that lower predictions are observed when a lower proportion of EUR is simulated as the sample size decreases, while this is compensated for by the additional information brought by non-EUR samples when there is at least genetic heterogeneity. Overall, we confirm that there is not one method with the best predictions in all scenarios

but confirm the advantage of trans-ancestry PGS in non-EUR populations from ancestry-diverse samples, including TAMR and CT-SLEB, using scenarios approximating the genetic architecture of these two traits. This highlights the relevance of these methods for compensating current bias in GWASs linked to unbalanced ancestry compositions.

Overall, genetic heterogeneity and its relationship with ancestry composition of the discovery sample have the greatest impact on the relative accuracy of the methods. This was observed both on theoretical scenarios and on scenarios approaching real human complex traits, T2D and TGs. Our results highlight the advantage of meta-analysis approaches that model ancestry-related genetic heterogeneity, such as meta-regression, which offers optimal accuracy levels. Nevertheless, even this method benefits from the inclusion of non-EUR individuals, especially if there is genetic heterogeneity underlying the trait of interest, emphasizing the need to include more diverse samples in future studies.

Our simulations were based on several simplifying assumptions to reduce computational burden. First, our simulations are based on independent variants to mimic a classical approach where PGS is built upon variants obtained from clumping and selection based on the p value of association.[34] We did not study the impact of LD, but we hypothesize that while it can have an impact on the absolute power of the methods, it will not modify the relative performance of the PGSs included in this study, as they were all built up only on the significant SNPs. We acknowledge the fact that many other PGS constructions exist, especially integrating all variants in the genome, but this comparison is beyond the scope of this work, which focuses on comparing the accuracy of meta-analysis methods. It is possible that genome-wide PGSs could have a better transferability to non-European populations, but we argue that the conclusions would remain similar in supporting the need to increase diversity at the study design in GWASs. Second, SNPs are all simulated as associated with the phenotype and we did not assess the impact of adding non-causal variants for computational reasons. The simulation of non-causal variants could result in lower performance compared with our simulations even if only a small fraction of these variants is expected to pass the significance threshold required for inclusion in the PGS. Third, only common variants have been considered in the traps simulation framework. Rare variants have also been shown to add predictive value of human complex traits[35] and tend to be most heterogeneously distributed among ancestries than common variants. The transferability of PGS to non-European populations in our simulations is therefore likely to be over-estimated, especially in the African populations. Fourth, for real trait scenarios, we estimate the parameters under the hypothesis that the full genetic architecture of TGs and T2D is known. Estimating the heterogeneity parameter is particularly hard, because the power to detect heterogeneous variants is itself limited by non-EUR sample size. It is therefore possible that the simulated heterogeneity is underestimated, which could lead to an overestimation of the performance in our simulations. Fifth, in our real-life scenarios, we extrapolate the number of additional causal SNPs required to explain the full narrow-sense heritability of the trait. We assumed this number grows linearly with heritability explained; however, we expect to identify variants with smaller effect sizes explaining a lower proportion of the phenotypic variance as sample sizes increase in future EUR-based GWASs. In non-EUR populations, Morales et al. showed that a higher number of genetic associations is also expected to emerge in future genetic studies from the inclusion of African and Hispanic populations due to their higher genetic diversity and lower LD.[36] Overall, while our simulation parameters may suffer from biases, we argue that although these biases might lead to an overestimation of the expected overall performance, they are not likely to impact the relative performance of the three PGS methods and the overall conclusion of this work. Moreover, all these limits highlight the difficulty in accurately estimating PGS predictions expected for a wide range of human complex traits, which highly depend on the underlying genetic architecture, and especially the heterogeneity of the trait. Finally, we assumed PGSs were transferrable at the population level. However, even within an ancestry-matched group, PGS predictions can be affected by sample variables such as the age or the sex of individuals,[37] or even at fine-scale within a population.[38] Our simulations are conducted using the five 1000 Genomes populations, but considering subpopulations would probably be needed to perform a more comprehensive evaluation. Veturi et al., for example, highlighted heterogeneity in effect sizes between European-Americans and African-Americans[39] and Kamiza et al. showed that PGS predictions greatly varied between the sub-Saharan and the Ugandan populations in Africa.[40] This assessment is limited by the use of the 1000 Genomes project itself for the simulations, as studies have shown that most subpopulations in the project actually represent the same underlying ancestry, especially in the SAS population.[41]

In summary, our results from simulations both on hypothetical and real trait scenarios show that PGS accuracy highly depends on the underlying genetic architecture of the trait, especially heterogeneity among populations. In traits with higher levels of genetic heterogeneity, TAMR PGS offers advantages compared with a fixed-effects meta-analysis or an AS score but the performances are still low given the current ancestry distribution. Predictions in non-European populations in this setting will also most probably benefit from the recent developments in trans-ancestry PGS given the results obtained with CT-SLEB in T2D scenarios. Our simulation results are in line with other studies highlighting the poor transferability of PGS to non-EUR populations[11,14] and support the urgent need to increase diversity in future genetic studies. Several large genetic projects are moving in that direction, such as the Uganda Genome Resource,[42] the H3Africa project,[43,44] or the GenomeAsia 100K project[45] focusing on specific large

ancestries, and the PAGE study[10] or the IHCC[46] integrating multi-continental ancestries. This translation will require new collaborations and a shift in the overall conception of genetic studies[5,47] but will undoubtedly lead to better comprehension of human complex traits. The translation to non-EUR populations represents the next step to widely accessible precision medicine[48] and is crucial to redress existing health disparities where using European-based data could lead to wrong conclusions in other populations.[13]

Data and code availability

The traps procedure is available at https://github.com/hmgu-itg/traps along with examples.

Supplemental information

Supplemental information can be found online at https://doi.org/10.1016/j.xhgg.2023.100214.

Acknowledgments

This project has received funding from the European Union's Horizon 2020 research and innovation program under Grant Agreement No 101017802 (OPTOMICS).

Declaration of interests

Arthur Gilly currently works at Regeneron Pharmaceuticals.

Received: January 27, 2023
Accepted: June 12, 2023

References

1. Buniello, A., MacArthur, J.A.L., Cerezo, M., Harris, L.W., Hayhurst, J., Malangone, C., McMahon, A., Morales, J., Mountjoy, E., Sollis, E., et al. (2019). The NHGRI-EBI GWAS Catalog of published genome-wide association studies, targeted arrays and summary statistics 2019. Nucleic Acids Res. *47*, D1005–D1012. https://doi.org/10.1093/nar/gky1120.
2. Sudlow, C., Gallacher, J., Allen, N., Beral, V., Burton, P., Danesh, J., Downey, P., Elliott, P., Green, J., Landray, M., et al. (2015). UK biobank: an open access resource for identifying the causes of a wide range of complex diseases of middle and old age. PLoS Med. *12*, e1001779. https://doi.org/10.1371/journal.pmed.1001779.
3. Taliun, D., Harris, D.N., Kessler, M.D., Carlson, J., Szpiech, Z.A., Torres, R., Taliun, S.A.G., Corvelo, A., Gogarten, S.M., Kang, H.M., et al. (2021). Sequencing of 53,831 diverse genomes from the NHLBI TOPMed Program. Nature *590*, 290–299. https://doi.org/10.1038/s41586-021-03205-y.
4. Kurki, M.I., Karjalainen, J., Palta, P., Sipilä, T.P., Kristiansson, K., Donner, K.M., Reeve, M.P., Laivuori, H., Aavikko, M., Kaunisto, M.A., et al. (2023). FinnGen provides genetic insights from a well-phenotyped isolated population. Nature *613*, 508–518. https://doi.org/10.1038/s41586-022-05473-8.
5. Ju, D., Hui, D., Hammond, D.A., Wonkam, A., and Tishkoff, S.A. (2022). Importance of Including Non-European Populations in Large Human Genetic Studies to Enhance Precision Medicine. Annu. Rev. Biomed. Data Sci. *5*, 321. https://doi.org/10.1146/annurev-biodatasci-339-112550.
6. Sirugo, G., Williams, S.M., and Tishkoff, S.A. (2019). The Missing Diversity in Human Genetic Studies. Cell *177*, 1080. https://doi.org/10.1016/j.cell.2019.04.032.
7. Wall, J.D., and Pritchard, J.K. (2003). Haplotype blocks and linkage disequilibrium in the human genome. Nat. Rev. Genet. *4*, 587–597. https://doi.org/10.1038/nrg1123.
8. Brown, B.C., Asian Genetic Epidemiology Network Type 2 Diabetes consortium, Ye, C.J., Price, A.L., and Zaitlen, N. (2016). Transethnic Genetic-Correlation Estimates from Summary Statistics. Am. J. Hum. Genet. *99*, 76–88. https://doi.org/10.1016/j.ajhg.2016.05.001.
9. Galinsky, K.J., Reshef, Y.A., Finucane, H.K., Loh, P.R., Zaitlen, N., Patterson, N.J., Brown, B.C., and Price, A.L. (2019). Estimating cross-population genetic correlations of causal effect sizes. Genet. Epidemiol. *43*, 180–188. https://doi.org/10.1002/gepi.22173.
10. Wojcik, G.L., Graff, M., Nishimura, K.K., Tao, R., Haessler, J., Gignoux, C.R., Highland, H.M., Patel, Y.M., Sorokin, E.P., Avery, C.L., et al. (2019). Genetic analyses of diverse populations improves discovery for complex traits. Nature *570*, 514–518. https://doi.org/10.1038/s41586-019-1310-4.
11. Duncan, L., Shen, H., Gelaye, B., Meijsen, J., Ressler, K., Feldman, M., Peterson, R., and Domingue, B. (2019). Analysis of polygenic risk score usage and performance in diverse human populations. Nat. Commun. *10*, 3328. https://doi.org/10.1038/s41467-019-11112-0.
12. Martin, A.R., Gignoux, C.R., Walters, R.K., Wojcik, G.L., Neale, B.M., Gravel, S., Daly, M.J., Bustamante, C.D., and Kenny, E.E. (2017). Human Demographic History Impacts Genetic Risk Prediction across Diverse Populations. Am. J. Hum. Genet. *100*, 635–649. https://doi.org/10.1016/j.ajhg.2017.03.004.
13. Martin, A.R., Kanai, M., Kamatani, Y., Okada, Y., Neale, B.M., and Daly, M.J. (2019). Clinical use of current polygenic risk scores may exacerbate health disparities. Nat. Genet. *51*, 584–591. https://doi.org/10.1038/s41588-019-0379-x.
14. Wang, Y., Guo, J., Ni, G., Yang, J., Visscher, P.M., and Yengo, L. (2020). Theoretical and empirical quantification of the accuracy of polygenic scores in ancestry divergent populations. Nat. Commun. *11*, 3865. https://doi.org/10.1038/s41467-020-17719-y.
15. Ding, Y., Hou, K., Xu, Z., Pimplaskar, A., Petter, E., Boulier, K., Privé, F., Vilhjálmsson, B.J., Loohuis, L.O., and Pasaniuc, B. (2022). Polygenic scoring accuracy varies across the genetic ancestry continuum in all human populations. Preprint at bioRxiv. https://doi.org/10.1101/2022.09.28.509988.
16. Curtis, D. (2018). Polygenic risk score for schizophrenia is more strongly associated with ancestry than with schizophrenia. Psychiatr. Genet. *28*, 85–89. https://doi.org/10.1097/YPG.0000000000000206.
17. Willer, C.J., Li, Y., and Abecasis, G.R. (2010). METAL: fast and efficient meta-analysis of genomewide association scans. Bioinformatics *26*, 2190–2191. https://doi.org/10.1093/bioinformatics/btq340.
18. Han, B., and Eskin, E. (2011). Random-effects model aimed at discovering associations in meta-analysis of genome-wide association studies. Am. J. Hum. Genet. *88*, 586–598. https://doi.org/10.1016/j.ajhg.2011.04.014.
19. Mägi, R., Horikoshi, M., Sofer, T., Mahajan, A., Kitajima, H., Franceschini, N., McCarthy, M.I., COGENT-Kidney Consortium T2D-GENES Consortium, and Morris, A.P. (2017). Trans-ethnic

meta-regression of genome-wide association studies accounting for ancestry increases power for discovery and improves fine--mapping resolution. Hum. Mol. Genet. *26*, 3639–3650. https://doi.org/10.1093/hmg/ddx280.
20. Márquez-Luna, C., Loh, P.R., South Asian Type 2 Diabetes SAT2D Consortium; and SIGMA Type 2 Diabetes Consortium, and Price, A.L. (2017). Multiethnic polygenic risk scores improve risk prediction in diverse populations. Genet. Epidemiol. *41*, 811–823. https://doi.org/10.1002/gepi.22083.
21. Weissbrod, O., Kanai, M., Shi, H., Gazal, S., Peyrot, W.J., Khera, A.V., Okada, Y., Biobank Japan Project, Martin, A.R., Finucane, H.K., and Price, A.L. (2022). Leveraging fine-mapping and multipopulation training data to improve cross-population polygenic risk scores. Nat. Genet. *54*, 450–458. https://doi.org/10.1038/s41588-022-01036-9.
22. Mahajan, A., Spracklen, C.N., Zhang, W., Ng, M.C.Y., Petty, L.E., Kitajima, H., Yu, G.Z., Rüeger, S., Speidel, L., Kim, Y.J., et al. (2022). Multi-ancestry genetic study of type 2 diabetes highlights the power of diverse populations for discovery and translation. Nat. Genet. *54*, 560–572. https://doi.org/10.1038/s41588-022-01058-3.
23. Cavazos, T.B., and Witte, J.S. (2021). Inclusion of variants discovered from diverse populations improves polygenic risk score transferability. HGG Adv. *2*, 100017. https://doi.org/10.1016/j.xhgg.2020.100017.
24. Ruan, Y., Lin, Y.F., Feng, Y.C.A., Chen, C.Y., Lam, M., Guo, Z., Stanley Global Asia Initiatives, He, L., Sawa, A., Martin, A.R., et al. (2022). Improving polygenic prediction in ancestrally diverse populations. Nat. Genet. *54*, 573–580. https://doi.org/10.1038/s41588-022-01054-7.
25. Koyama, S., Ito, K., Terao, C., Akiyama, M., Horikoshi, M., Momozawa, Y., Matsunaga, H., Ieki, H., Ozaki, K., Onouchi, Y., et al. (2020). Population-specific and trans-ancestry genome-wide analyses identify distinct and shared genetic risk loci for coronary artery disease. Nat. Genet. *52*, 1169–1177. https://doi.org/10.1038/s41588-020-0705-3.
26. Udler, M.S., McCarthy, M.I., Florez, J.C., and Mahajan, A. (2019). Genetic Risk Scores for Diabetes Diagnosis and Precision Medicine. Endocr. Rev. *40*, 1500–1520. https://doi.org/10.1210/er.2019-00088.
27. Sudmant, P.H., Rausch, T., Gardner, E.J., Handsaker, R.E., Abyzov, A., Huddleston, J., Zhang, Y., Ye, K., Jun, G., Fritz, M.H.Y., et al. (2015). An integrated map of structural variation in 2,504 human genomes. Nature *526*, 75–81. https://doi.org/10.1038/nature15394.
28. O'Connor, L.J. (2021). The distribution of common-variant effect sizes. Nat. Genet. *53*, 1243–1249. https://doi.org/10.1038/s41588-021-00901-3.
29. Graham, S.E., Clarke, S.L., Wu, K.H.H., Kanoni, S., Zajac, G.J.M., Ramdas, S., Surakka, I., Ntalla, I., Vedantam, S., Winkler, T.W., et al. (2021). The power of genetic diversity in genome-wide association studies of lipids. Nature *600*, 675–679. https://doi.org/10.1038/s41586-021-04064-3.
30. Blackburn, N.B., Porto, A., Peralta, J.M., and Blangero, J. (2018). Heritability and genetic associations of triglyceride and HDL-C levels using pedigree-based and empirical kinships. BMC Proc. *12*, 34. https://doi.org/10.1186/s12919-018-0133-x.
31. Almgren, P., Lehtovirta, M., Isomaa, B., Sarelin, L., Taskinen, M.R., Lyssenko, V., Tuomi, T., Groop, L.; and Botnia Study Group (2011). Heritability and familiality of type 2 diabetes and related quantitative traits in the Botnia Study. Diabetologia *54*, 2811–2819. https://doi.org/10.1007/s00125-011-2267-5.
32. Huang, Q.Q., Sallah, N., Dunca, D., Trivedi, B., Hunt, K.A., Hodgson, S., Lambert, S.A., Arciero, E., Wright, J., Griffiths, C., et al. (2022). Transferability of genetic loci and polygenic scores for cardiometabolic traits in British Pakistani and Bangladeshi individuals. Nat. Commun. *13*, 4664. https://doi.org/10.1038/s41467-022-32095-5.
33. Zhang, H., Zhan, J., Jin, J., Zhang, J., Lu, W., Zhao, R., Ahearn, T.U., Yu, Z., O'Connell, J., Jiang, Y., et al. (2023). Novel Methods for Multi-ancestry Polygenic Prediction and their Evaluations in 5.1 Million Individuals of Diverse Ancestry. Preprint at bioRxiv. https://doi.org/10.1101/2022.03.24.485519.
34. Choi, S.W., Mak, T.S.H., and O'Reilly, P.F. (2020). Tutorial: a guide to performing polygenic risk score analyses. Nat. Protoc. *15*, 2759–2772. https://doi.org/10.1038/s41596-020-0353-1.
35. Dornbos, P., Koesterer, R., Ruttenburg, A., Nguyen, T., Cole, J.B., AMP-T2D-GENES Consortium, Leong, A., Meigs, J.B., Florez, J.C., Rotter, J.I., et al. (2022). A combined polygenic score of 21,293 rare and 22 common variants improves diabetes diagnosis based on hemoglobin A1C levels. Nat. Genet. *54*, 1609–1614. https://doi.org/10.1038/s41588-022-01200-1.
36. Morales, J., Welter, D., Bowler, E.H., Cerezo, M., Harris, L.W., McMahon, A.C., Hall, P., Junkins, H.A., Milano, A., Hastings, E., et al. (2018). A standardized framework for representation of ancestry data in genomics studies, with application to the NHGRI-EBI GWAS Catalog. Genome Biol. *19*, 21. https://doi.org/10.1186/s13059-018-1396-2.
37. Robette, N., Génin, E., and Clerget-Darpoux, F. (2022). Heritability: What's the point? What is it not for? A human genetics perspective. Genetica *150*, 199–208. https://doi.org/10.1007/s10709-022-00149-7.
38. Mostafavi, H., Harpak, A., Agarwal, I., Conley, D., Pritchard, J.K., and Przeworski, M. (2020). Variable prediction accuracy of polygenic scores within an ancestry group. Elife *9*, e48376. https://doi.org/10.7554/eLife.48376.
39. Veturi, Y., de Los Campos, G., Yi, N., Huang, W., Vazquez, A.I., and Kühnel, B. (2019). Modeling Heterogeneity in the Genetic Architecture of Ethnically Diverse Groups Using Random Effect Interaction Models. Genetics *211*, 1395–1407. https://doi.org/10.1534/genetics.119.301909.
40. Kamiza, A.B., Toure, S.M., Vujkovic, M., Machipisa, T., Soremekun, O.S., Kintu, C., Corpas, M., Pirie, F., Young, E., Gill, D., et al. (2022). Transferability of genetic risk scores in African populations. Nat. Med. *28*, 1163–1166. https://doi.org/10.1038/s41591-022-01835-x.
41. Sengupta, D., Choudhury, A., Basu, A., and Ramsay, M. (2016). Population Stratification and Underrepresentation of Indian Subcontinent Genetic Diversity in the 1000 Genomes Project Dataset. Genome Biol. Evol. *8*, 3460–3470. https://doi.org/10.1093/gbe/evw244.
42. Gurdasani, D., Carstensen, T., Fatumo, S., Chen, G., Franklin, C.S., Prado-Martinez, J., Bouman, H., Abascal, F., Haber, M., Tachmazidou, I., et al. (2019). Uganda Genome Resource Enables Insights into Population History and Genomic Discovery in Africa. Cell *179*, 984–1002.e36. https://doi.org/10.1016/j.cell.2019.10.004.
43. H3Africa Consortium, Rotimi, C., Abayomi, A., Abimiku, A., Adabayeri, V.M., Adebamowo, C., Adebiyi, E., Ademola, A.D., Adeyemo, A., Adu, D., et al. (2014). Research capacity.

Enabling the genomic revolution in Africa. Science *344*, 1346–1348. https://doi.org/10.1126/science.1251546.

44. Mulder, N., Abimiku, A., Adebamowo, S.N., de Vries, J., Matimba, A., Olowoyo, P., Ramsay, M., Skelton, M., and Stein, D.J. (2018). H3Africa: current perspectives. Pharmgenomics. Pers. Med. *11*, 59–66. https://doi.org/10.2147/PGPM.S141546.
45. GenomeAsia100K Consortium (2019). The GenomeAsia 100K Project enables genetic discoveries across Asia. Nature *576*, 106–111. https://doi.org/10.1038/s41586-019-1793-z.
46. Manolio, T.A., Goodhand, P., and Ginsburg, G. (2020). The International Hundred Thousand Plus Cohort Consortium: integrating large-scale cohorts to address global scientific challenges. Lancet. Digit. Health *2*, e567–e568. https://doi.org/10.1016/S2589-7500(20)30242-9.
47. Fatumo, S., Chikowore, T., Choudhury, A., Ayub, M., Martin, A.R., and Kuchenbaecker, K. (2022). A roadmap to increase diversity in genomic studies. Nat. Med. *28*, 243–250. https://doi.org/10.1038/s41591-021-01672-4.
48. Popejoy, A.B., and Fullerton, S.M. (2016). Genomics is failing on diversity. Nature *538*, 161–164. https://doi.org/10.1038/538161a.

HGGA, Volume *4*

Supplemental information

Bridging the diversity gap: Analytical and study design considerations for improving the accuracy of trans-ancestry genetic prediction

Ozvan Bocher, Arthur Gilly, Young-Chan Park, Eleftheria Zeggini, and Andrew P. Morris

Supplemental Figures

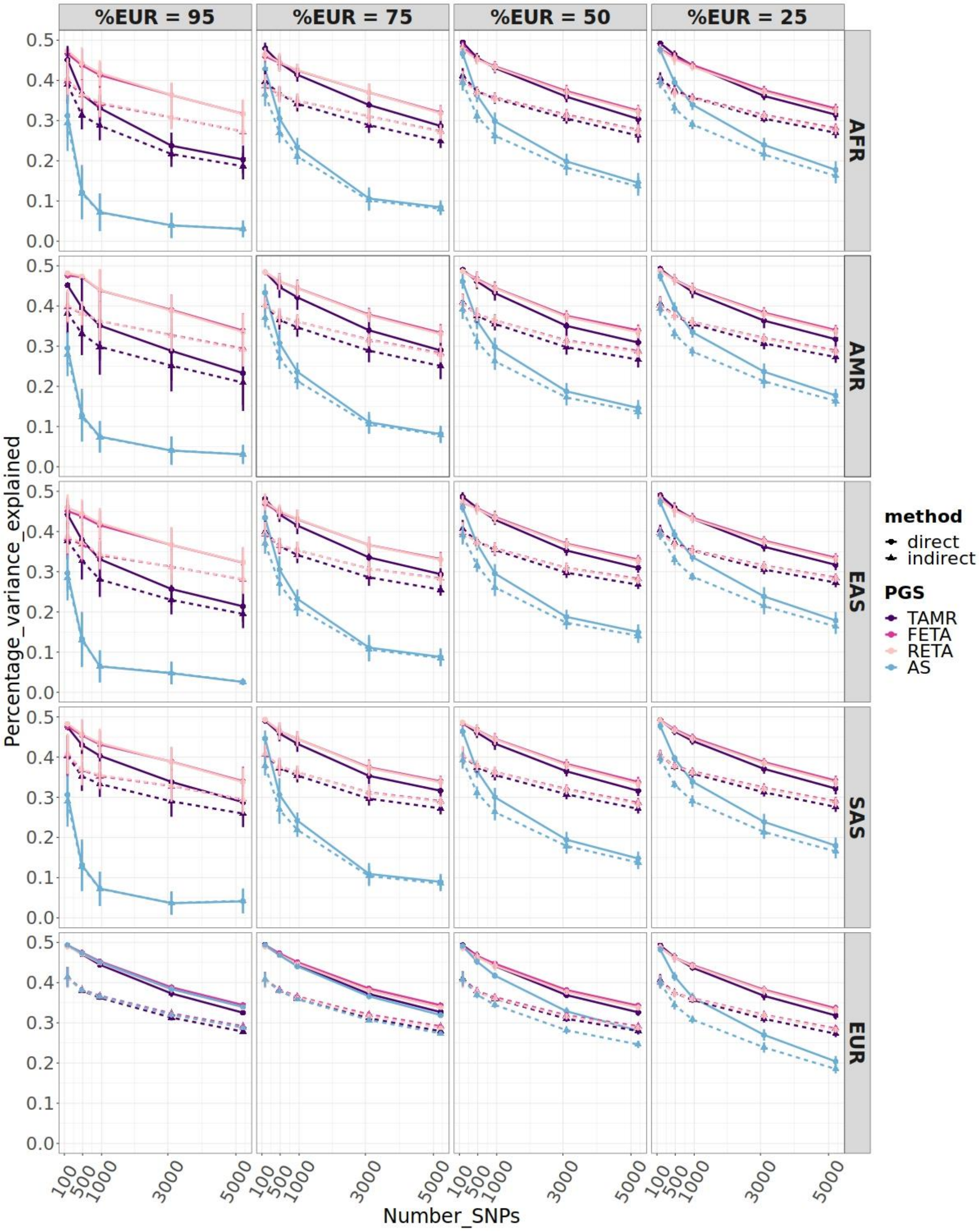

Figure S1: Assessment of the impact of the number of causal SNPs on the accuracy of the three PGS TAMR, FETA and AS. A heritability of 50% was simulated with no heterogeneity. PGS accuracies were evaluated in the five 1000 Genomes populations for four percentages of European-ancestry individuals. Error bars represent the standard error of the mean variance explained across 10 simulation replicates.

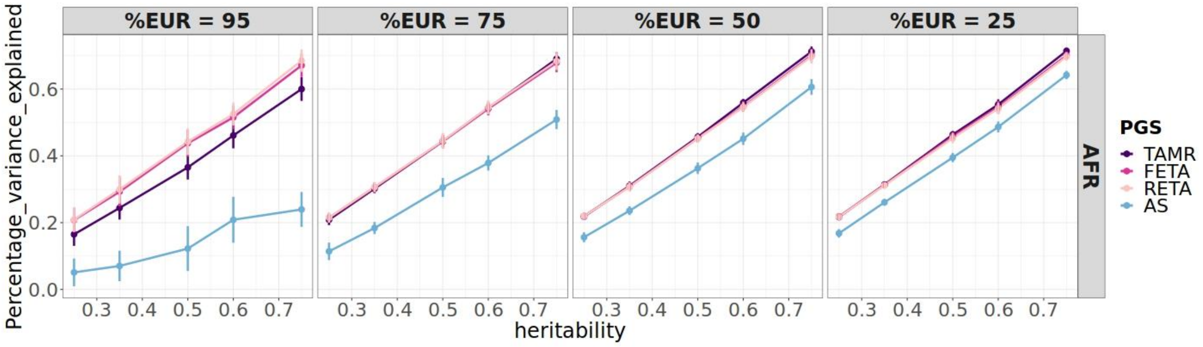

Figure S2: Assessment of the impact of the simulated heritability on the accuracy of the three PGS TAMR, FETA and AS. 500 causal SNPs were simulated with no heterogeneity. PGS accuracies were evaluated in the African population from the 1000 Genome project for four percentages of European-ancestry individuals. Error bars represent the standard error of the mean variance explained across 10 simulation replicates.

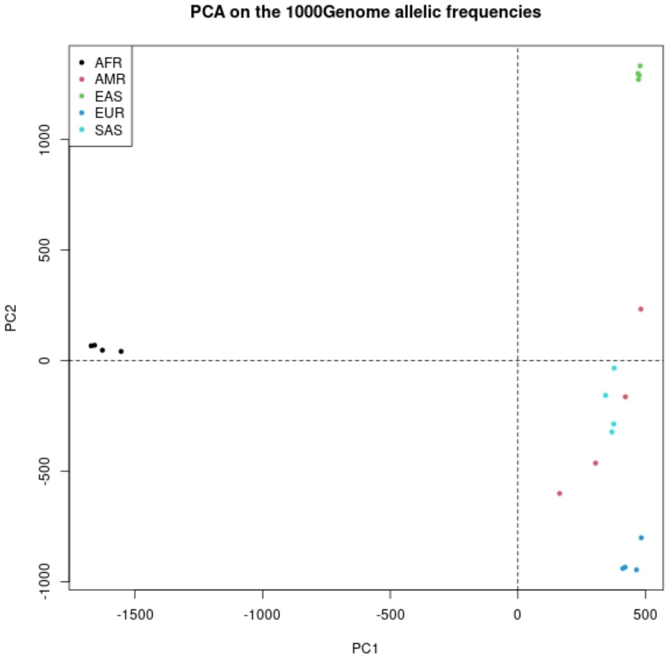

Figure S3: PCA on the allelic frequencies from 20 subpopulations from the 1000Genomes project used for the simulations

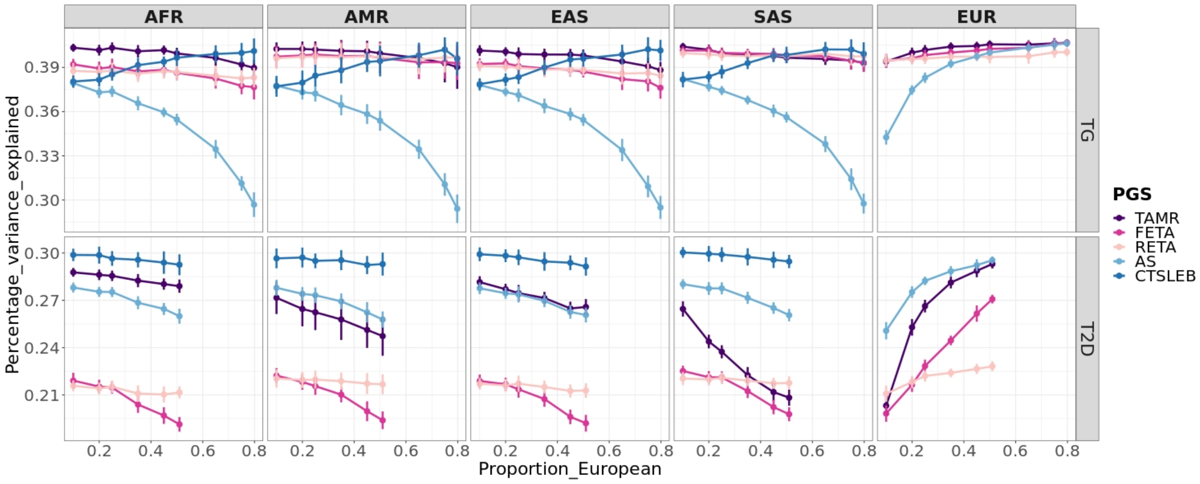

Supplementary Figure 4: Evaluation of five PGS constructions for two human complex traits, Triglycerides and Type 2 Diabetes, in the five 1000Genomes populations. Non-European populations are simulated in equal sample sizes. Error bars represent the standard error of the mean variance explained across 10 simulation replicates. This figure corresponds to Figure 3 in the main text with the additional method CT-SLEB being tested.

Supplemental tables

		TG	T2D
**Original study**	Study	10.1038/s41586-021-04064-3	10.1038/s41588-022-01058-3
	Number of independent SNP	461	338
	Variance explained (%)	9	8
	Sample size	1,654,960	1,339,889
	EUR/AFR/AMR/EAS/SAS (%)	79.8/6/2.9/8.8/2.5	51.1/6.6/5.6/28.4/8.3
	Heterogeneity (%)	19	40
**Simulations**	Sample size	1,654,960	1,339,889
	EUR/AFR/AMR/EAS/SAS (%)	79.8/5.3 (x4)<br>75/6.25 (x4)<br>65/8.75 (x4)<br>50/12.5 (x4)<br>45/13.75 (x4)<br>35/16.25 (x4)<br>25/18.75 (x4)<br>20 (x5)<br>10/22.5 (x4)	51.1/12.225 (x4)<br>45/13.75 (x4)<br>35/16.25 (x4)<br>25/18.75 (x4)<br>20 (x5)<br>10/22.5 (x4)
	Heterogeneity (%)	1	30
	Heritability (%)	42	31
	Number of independent SNP	2151	1310

Table S1: Parameters estimated from two studies on TGs and T2D and the corresponding values in the traps simulations.

Supplemental methods

Overview of the pipeline

We have developed a pipeline for TRans Ancestry PGS Simulation (traps, https://github.com/hmgu-itg/traps) that simulates genetic data using 1000Genomes project allelic frequencies[1], performs GWAS, constructs PGS and assesses their performance in multiple ancestry groups. We randomly choose four subpopulations in each of the five 1000Genomes population groups: AFR (GWD, LWK, MSL, YRI), AMR (CLM, MXL, PEL, PUR), EAS (CDX, CHB, JPT, KHV), EUR (FIN, GBS, TSI, IBS) and SAS (BEB, GIH, PJL, STU). We then simulate genetic data and perform association tests within each of the 20 subpopulations. The different steps of traps are detailed hereafter.

Simulations

First, we simulate genetic data for a user-specified number of genetic variants sampled from the common variants observed in the 1000Genomes project (overall minor allele frequency or 'MAF' higher than 1%). Genotypes are simulated for individuals in each subpopulation under the Hardy-Weinberg equilibrium using the allelic frequencies observed in the corresponding 1000Genomes subpopulation (drawn genetic variants are considered independent). The sample's ancestry composition, expressed either as a proportion of Europeans with the remaining population equally distributed, or five ancestry-specific proportions summing to one, is specified by the user. Unless otherwise stated, 150,000 individuals are simulated in this work, and the first method is used to specify ancestry composition.

Second, we simulate an associated phenotype by drawing a genetic effect w at random for each variant (note that all simulated variants are considered as causal) according to the log-normal model[2] with $log10(w) \sim N(-log10(M) - 0.8^2, 0.8)$, M representing the total number of causal variants.

These effects are then normalized into units of phenotype standard deviation per unit of genotype standard deviation, and further adjusted so that the sum of squared effect sizes is equal to a user-specified heritability value (h^2_{SNP}) as: $\beta_s = \sqrt{\frac{h^2}{\sum_{s=1}^{S} w_s}}$. Finally, continuous phenotypes are simulated as: $y = \beta G + \varepsilon$ where β are the normalized effect sizes of the SNPs, G the corresponding genotypes and $\varepsilon \sim N\left(0, \sqrt{1 - h^2_{SNP}}\right)$.

We apply this simulation procedure once for generating a "base" dataset used for PGS construction, and once for a "target" dataset used in PGS evaluation.

Association analysis

After simulating genotypes and phenotypes, we perform association tests in each of the 20 subpopulations in the base and in the target samples separately using the PLINK software[3]. Three meta-analyses are then performed to obtain SNPs weights for combined PGS:

1. Fixed-Effect Trans-Ancestry (FETA) which is a classical fixed-effect cross-ancestry meta-analysis across all subpopulations using the METASOFT software[4].
2. Random-Effect Trans-Ancestry (RETA) which is a classical random-effect cross-ancestry meta-analysis across all subpopulations using the RE2 model from the METASOFT software[4]
3. Trans-Ancestry Meta-Regression (TAMR) which applies the meta-regression from Magi et al.[5] where axes of genetic variation representing the ancestry of the individuals model the heterogeneity related to ancestry between the effects in the different subpopulations. These axes of genetic variation are integrated along with estimates from GWAS in the base sample

to produce one adjusted effect per subpopulation. In traps, we use the first three principal components of the PCA on the 1000Genomes project allelic frequencies for this adjustment.

PGS construction and evaluation

SNP effect sizes from the meta-analysis serve as weights for the corresponding PGS which are then applied to the 20 target subpopulations. For FETA and RETA, the same effects from the trans-ancestry meta-analysis are applied to all samples irrespective of their origin. For TAMR, ancestry-adjusted effects computed in each subpopulation are used to compute PGS. We select SNPs with a p-value lower than $5x10^{-8}$ in the base sample to mimic commonly used PGS approaches[6]. In addition to the two meta-analysis PGS, an ancestry-specific (AS) PGS is computed in every population using the same significance threshold and corresponding SNP weights from GWAS in the base sample.

We evaluate the three PGS using either a 'direct' or an 'indirect' method. In the direct method, we suppose that individual data are available and PGS are computed for each individual *j* as: $PGS_j = \sum_{s=1}^{S} \beta_s G_{j,s}$. For quantitative traits, PGS_j is the predicted phenotype, while for binary traits, it is the predicted genetic liability. R^2 is used as a measure of fit. When only summary statistics are available, goodness of fit can be measured indirectly by:

$$1 - \exp\left(\left[\frac{\sum_{s=1}^{S} \beta_{b,s} \times \beta_{t,s} \times sd_{t,s}^{-2}}{\sum_{s=1}^{S} \beta_{t,s} \times sd_{t,s}{}^{-2}} \times \frac{1}{\sqrt{\frac{1}{\beta_{t,s}^{2} \times sd_{t,s}{}^{-2}}}}\right]^2 \times \frac{1}{n}\right)$$

With β_b and β_t the estimated effects in the base and target samples respectively, sd_t the corresponding standard deviation and *n* the sample size of the target sample. More details can be found in Dastani et al.[7] and have been implemented in the gtx R package[8]. Finally, we average R^2 across subpopulations to produce one measure of fit per ancestry group.

Simulated scenarios

General comparisons of parameters

We simulate 56 scenarios corresponding to a grid where we varied the following parameters: (1) the number of SNPs sampled from the 1000Genomes allele frequencies; (2) the heritability of the trait on which SNP effects are adjusted; (3) the proportion of European-ancestry individuals; (4) the percentage of SNPs with population-specific genetic effects. For each scenario, we perform 10 replicates and average accuracy.

Scenarios approximating real traits

Second, we simulate data under real-world scenarios for two complex traits that have been recently investigated in large trans-ancestry studies: type 2 diabetes[9] (T2D) for which we simulate underlying liability, and triglycerides levels[10] (TGs). Since our objective is to evaluate the ideal ancestry composition of future GWAS, we assume that these simulations capture the full genetic architecture of the traits. We estimate the expected number of associated SNPs as: $M = M_d * \frac{h^2}{h_d^2}$ with M_d corresponding to the number of independent SNPs associated at genome-wide significance in these two studies, h_d^2 the cumulative percentage of variance they explain and h^2 the heritability of the trait estimated from family-based studies. The heritability was estimated in previous studies as 0.42 for TGs[11] and 0.31 for T2D[12]. The proportion of heterogeneity was estimated in each study as the proportion of SNPs having a nominally significant p-value of heterogeneity due to ancestry, as

provided by MR-MEGA. We selected the proportion of heterogeneous SNPs in our simulations such that, across simulated data sets, the estimated proportion of SNPs with nominal evidence (P<0.05) of ancestry-correlated heterogeneity matched that observed in the real data. Finally, we assess PGS predictions using increasingly diverse sample from the original studies. We present figures from the two studies and the corresponding parameter values for the simulations in Table S1.

Supplemental references

1 Sudmant, P. H. *et al.* An integrated map of structural variation in 2,504 human genomes. *Nature* **526**, 75-81 (2015). https://doi.org:10.1038/nature15394

2 O'Connor, L. J. The distribution of common-variant effect sizes. *Nat Genet* **53**, 1243-1249 (2021). https://doi.org:10.1038/s41588-021-00901-3

3 Purcell, S. *et al.* PLINK: a tool set for whole-genome association and population-based linkage analyses. *Am J Hum Genet* **81**, 559-575 (2007). https://doi.org:10.1086/519795

4 Han, B. & Eskin, E. Random-effects model aimed at discovering associations in meta-analysis of genome-wide association studies. *Am J Hum Genet* **88**, 586-598 (2011). https://doi.org:10.1016/j.ajhg.2011.04.014

5 Magi, R. *et al.* Trans-ethnic meta-regression of genome-wide association studies accounting for ancestry increases power for discovery and improves fine-mapping resolution. *Hum Mol Genet* **26**, 3639-3650 (2017). https://doi.org:10.1093/hmg/ddx280

6 Choi, S. W., Mak, T. S. & O'Reilly, P. F. Tutorial: a guide to performing polygenic risk score analyses. *Nat Protoc* **15**, 2759-2772 (2020). https://doi.org:10.1038/s41596-020-0353-1

7 Dastani, Z. *et al.* Novel loci for adiponectin levels and their influence on type 2 diabetes and metabolic traits: a multi-ethnic meta-analysis of 45,891 individuals. *PLoS Genet* **8**, e1002607 (2012). https://doi.org:10.1371/journal.pgen.1002607

8 gtx (R CRAN, 2019).

9 Mahajan, A. *et al.* Multi-ancestry genetic study of type 2 diabetes highlights the power of diverse populations for discovery and translation. *Nat Genet* **54**, 560-572 (2022). https://doi.org:10.1038/s41588-022-01058-3

10 Graham, S. E. *et al.* The power of genetic diversity in genome-wide association studies of lipids. *Nature* **600**, 675-679 (2021). https://doi.org:10.1038/s41586-021-04064-3

11 Blackburn, N. B., Porto, A., Peralta, J. M. & Blangero, J. Heritability and genetic associations of triglyceride and HDL-C levels using pedigree-based and empirical kinships. *BMC Proc* **12**, 34 (2018). https://doi.org:10.1186/s12919-018-0133-x

12 Almgren, P. *et al.* Heritability and familiality of type 2 diabetes and related quantitative traits in the Botnia Study. *Diabetologia* **54**, 2811-2819 (2011). https://doi.org:10.1007/s00125-011-2267-5
